# Supplementary material for: METTL3/METTL14 maintain human nucleoli integrity by mediating SUV39H1/H2 degradation
Source: Nat Commun. 2024 Aug 21;15:7186. doi: 10.1038/s41467-024-51742-7 (PMC11339338; doi:10.1038/s41467-024-51742-7)

SUPPLEMENTARY INFORMATION

METTL3/METTL14 maintain human nucleoli integrity by mediating SUV39H1/H2 degradation

Supplementary information includes:

Supplementary Figure 1-8.

Supplementary Table 1. List of sgRNAs and primers for gene targeting.

Supplementary Table 2. List of primers for qRT-PCR.

Supplementary Table 3. List of antibodies used in this study.

Supplementary Table 4. List of primer sequences for over-expression.

Uncropped gel and blots.

Supplementary Figure 1

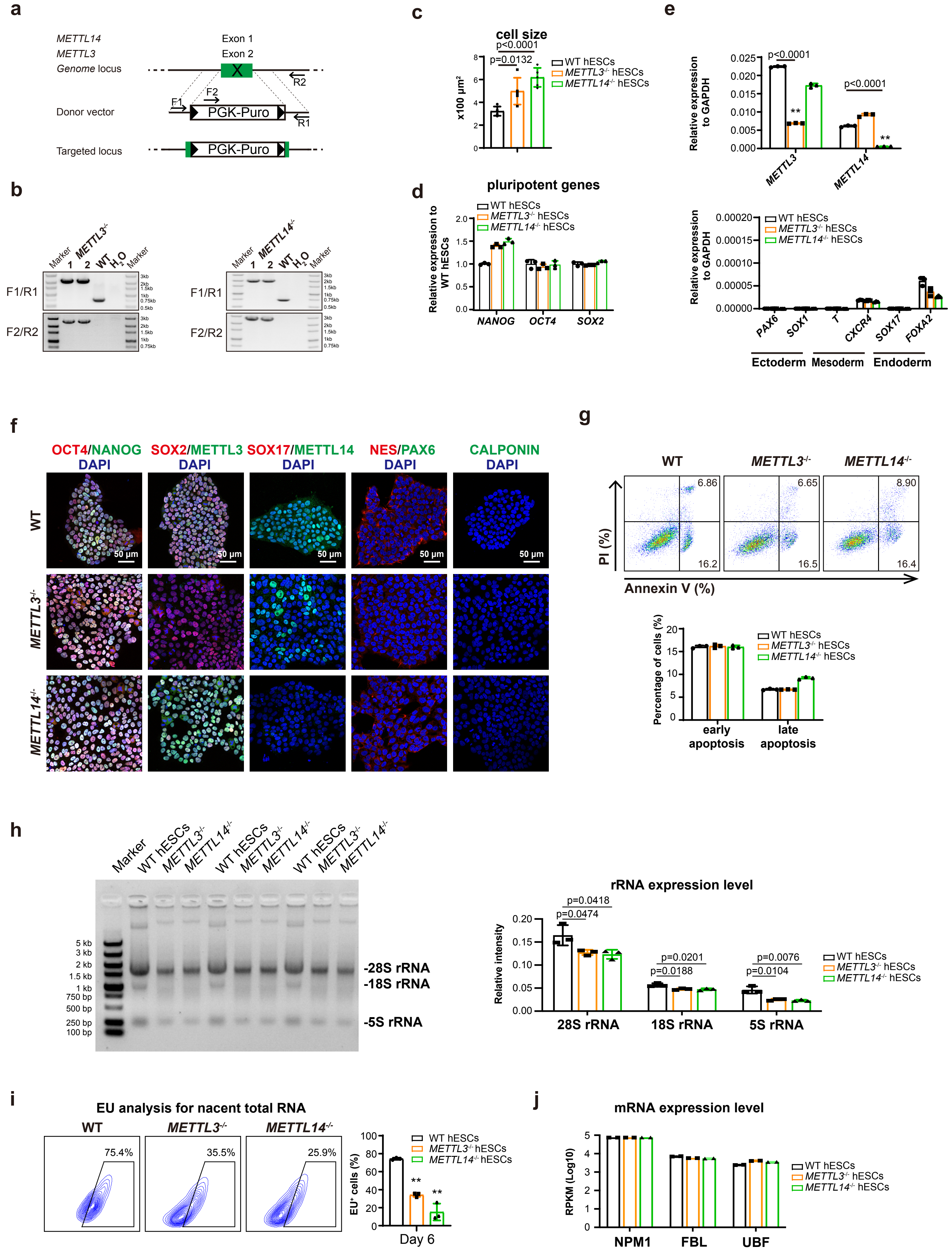

Supplementary Figure 1. Knockout of *METTL3* and *METTL14* in human ESCs

(a) Overview of the gene targeting strategy for *METTL3* and *METTL14*. **(b)** PCR identification for targeted deletion of *METTL3* and *METTL14*, respectively. **(c)** The quantification of cell size in the indicated cells. The data represent mean ± SD from five independent experiments (n=5). Statistical analysis, unpaired two-tailed Student's t-test. **(d-e)** qRT-PCR analysis on the expression level of pluripotent genes *NANOG*/*OCT4*/*SOX2*, *METTL3*/*METTL14*, ectoderm genes PAX6/*SOX1*, mesoderm genes *T*/*CXCR4*, endoderm genes SOX17/FOXA2 in the indicated cells. The data represent mean ± SD from three independent experiments. Statistical analysis, unpaired two-tailed Student's t-test. **(f)** Immunostaining on the pluripotent markers OCT4/SOX2/NANOG, three germ-layer markers NES/PAX6/CALPONIN/SOX17, METTL3, and METTL14 in the indicated cells. Scale bar, 50 μm. **(g)** Apoptosis analysis in the indicated cells. PI- and/or annexin V-positive cells were analyzed by FACS. The data represent mean ± SD from three independent experiments. Statistical analysis, unpaired two-tailed Student's t-test. **(h)** Total RNA electrophoresis from 0.5 million cells and corresponding rRNA quantification results. The data represent mean ± SD from three independent experiments (n=3). Statistical analysis, unpaired two-tailed Student's t-test. **(i)** FACS for EU analysis for nascent total RNA and their quantification results in the indicated cells. The data represent mean ± SD from three independent experiments. Statistical analysis, unpaired two-tailed Student's t-test. **(j)** the expression level of the nucleolar markers NPM1, FBL, and UBF from RNA-seq results in the indicated cell lines.

Supplementary Figure 2

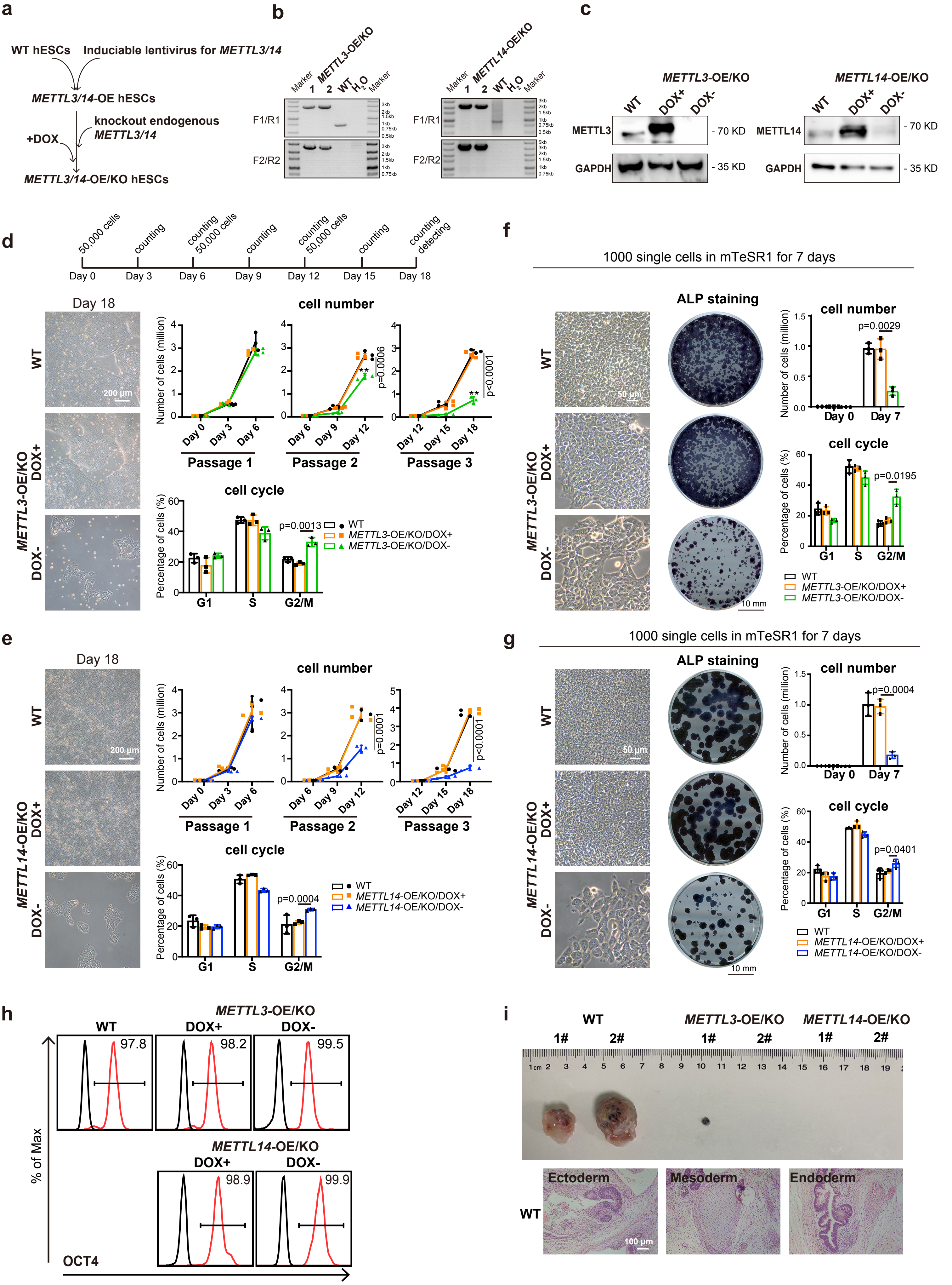

Supplementary Figure 2. Inducible knockout of *METTL3* and *METTL14* in human ESCs

(a) Strategy for inducible knockout of *METTL3* or *METTL14* in wild type (WT) H1 hESCs. **(b)** PCR identification for inducible targeted deletion of *METTL3* and *METTL14*, respectively. **(c)** Western blot analysis for METTL3 and METTL14 in the indicated cells. *METTL3*-OE/KO/DOX+ (*METTL3*-rescued), *METTL3*-OE/KO/DOX- (*METTL3*-KO); *METTL14*-OE/KO/DOX+ (*METTL14*-rescued), *METTL14*-OE/KO/DOX- (*METTL14*-deficient). **(d-e)** Morphology, continuous cell count analysis, and cell cycle analysis in the indicated cells. Scale bar, 200 μm. **(f-g)** 1000 single cells were cultured for 7 days. A series of analysis including morphology, alkaline phosphatase (ALP) staining, cell number, and cell cycle analysis in the indicated cells. Scale bar, 50 μm and 10 mm. **(d-g)** The data represent mean ± SD from three independent experiments. Statistical analysis, unpaired two-tailed Student's t-test. **(h)** FACS analysis for pluripotency marker OCT4 in the indicated cells. **(i)** Teratoma formation analysis for the indicated cell lines. H&E staining on sections of teratomas formed by Wild type H1 hESCs. Scale bar, 100 μm.

Supplementary Figure 3

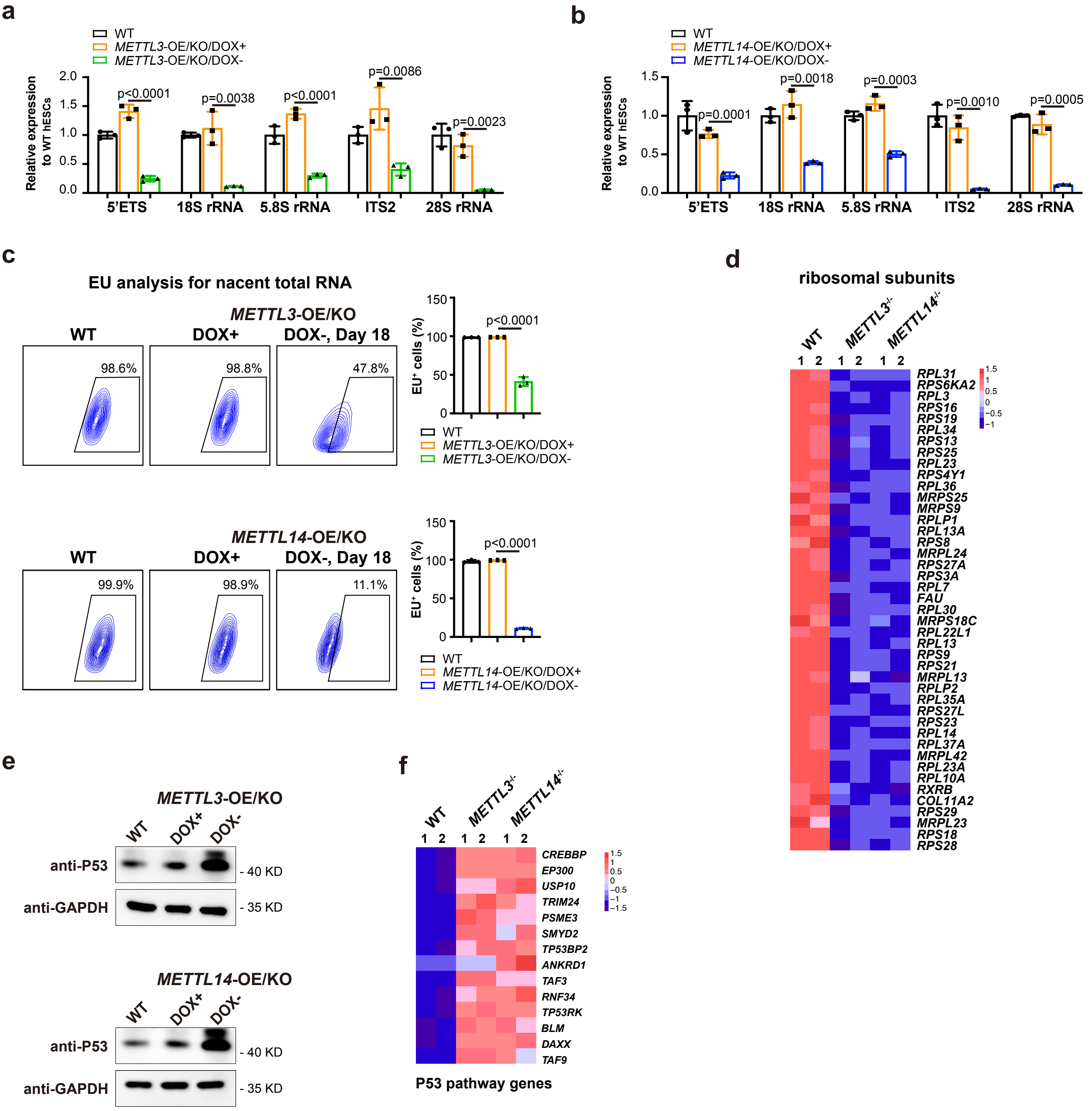

Supplementary Figure 3. Deficiency of *METTL3* and *METTL14* leads to nucleolar stress in human ESCs

(a-b) qRT-PCR analysis on the expression level of pre-rRNA in the indicated cell lines. **(c)** FACS for EU analysis for nascent total RNA and their quantification results in the indicated cell lines. **(a-c)** The data represent mean ± SD from three independent experiments. Statistical analysis, unpaired two-tailed Student's t-test. **(d)** Heatmap on the expressions of ribosomal subunits in the indicated cell lines. **(e)** Western blot analysis on P53 protein in the indicated cells. **(f)** Heatmap analysis on P53 pathway genes in wild type (WT), *METTL3*- and *METTL14*-deficient hESCs.

Supplementary Figure 4

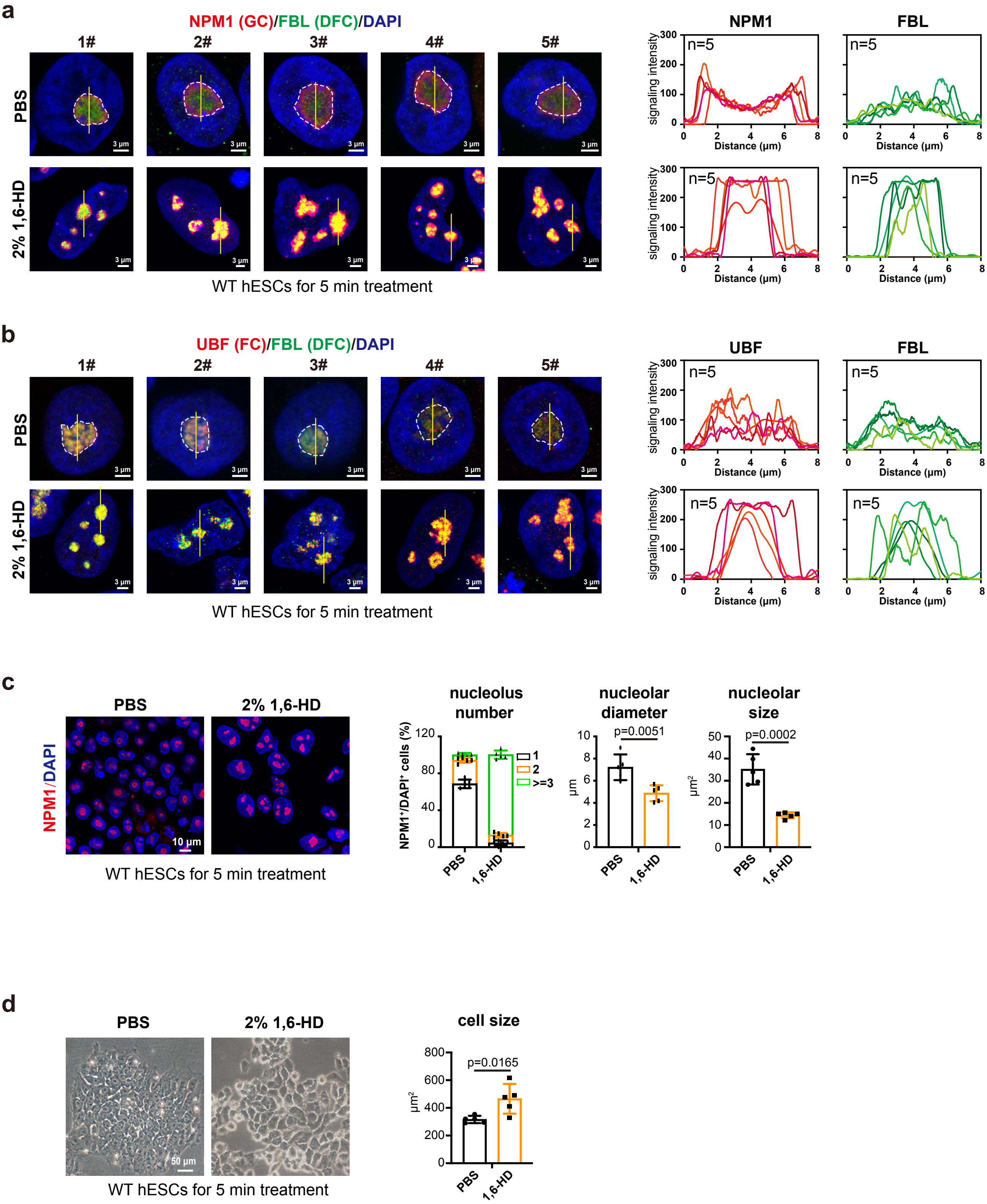

Supplementary Figure 4. 1,6 HD treatment phenocopies the nucleolar defect in *METTL3*- and *METTL14*-deficient hESCs

(a) Five representative immunostaining on FBL (DFC marker) and NPM1 (GC marker) in wild type (WT) H1 hESCs with 1,6 HD treatment for 5 minutes. Scale bar, 3 μm. Right panel, line scans depict signaling strength of NPM1 (red) and FBL (green) across nucleolus. **(b)** Five representative immunostaining on FBL (DFC marker) and UBF (FC marker) in wild type (WT) H1 hESCs with 1,6 HD treatment and PBS for 5 minutes, respectively. Scale bar, 3 μm. Right panel, line scans depict signaling strength of UBF (red) and FBL (green) across nucleolus. **(c)** Upper panel, immunostaining on NPM1 in WT hESCs with 1,6 HD treatment for 5 minutes. Scale bar, 10 μm and 3 μm. Lower panel, the quantification of nucleolus number, nucleolar diameter and size in WT hESCs with 1,6 HD treatment. **(d)** Morphology and the quantification of cell size of WT hESCs with 1,6 HD treatment. Scale bar, 50 μm. **(c-d)** The data represent mean ± SD from five independent experiments (n=5). Statistical analysis, unpaired two-tailed Student's t-test.

Supplementary Figure 5

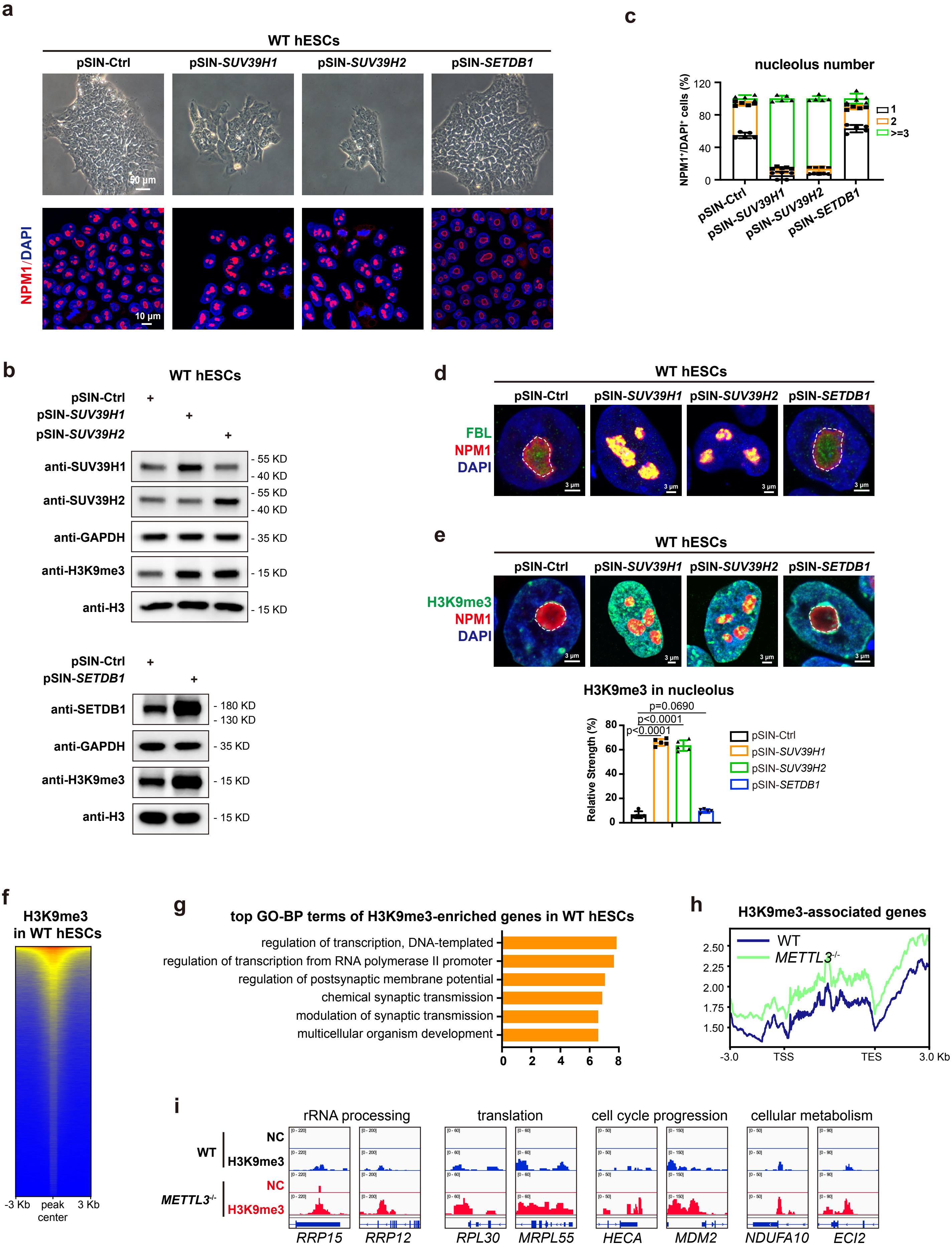

Supplementary Figure 5. Infiltration of heterochromatin in nucleoli in METTL3/METTL14-deficient hESCs

(a) Morphology immunostaining on the nucleolus marker NPM1 in *SUV39H1*-, *SUV39H2*-, and *SETDB1*- overexpressed hESCs, respectively. **(b)** Western blot analysis for SUV39H1/2, SETDB1, and H3K9me3 in the indicated cells in **(a)**. **(c)** The quantification of nucleolus number in the indicated cell lines. **(d)** Immunostaining on FBL and NPM1 in the indicated cell lines in **(a)**. Scale bar, 3 μm. (**e)** Immunostaining analysis on H3K9me3 and NPM1 and quantification for H3K9me3 in nucleolus in the indicated cells in **(a)**. Scale bar, 3 μm. **(f)** Heatmap of CUT&TAG-seq data from H3K9me3-associated peaks in wild-type (WT) hESCs. **(g)** GO analysis of H3K9me3-binding genes in WT hESCs. **(h)** Signal densities of H3K9me3-associated genes in the wild-type (WT) or *METTL3*$^{-/-}$ cells from CUT&TAG-seq data. **(i)** Genomic views of the H3K9me3 binding of genes related to rRNA processing, translation, cell cycle progression as well as cellular metabolism in the indicated cell lines. The data in **Supplementary Fig. 5c and 5e** represent mean ± SD from five independent experiments (n=5). Statistical analysis, unpaired two-tailed Student's t-test.

Supplementary Figure 6

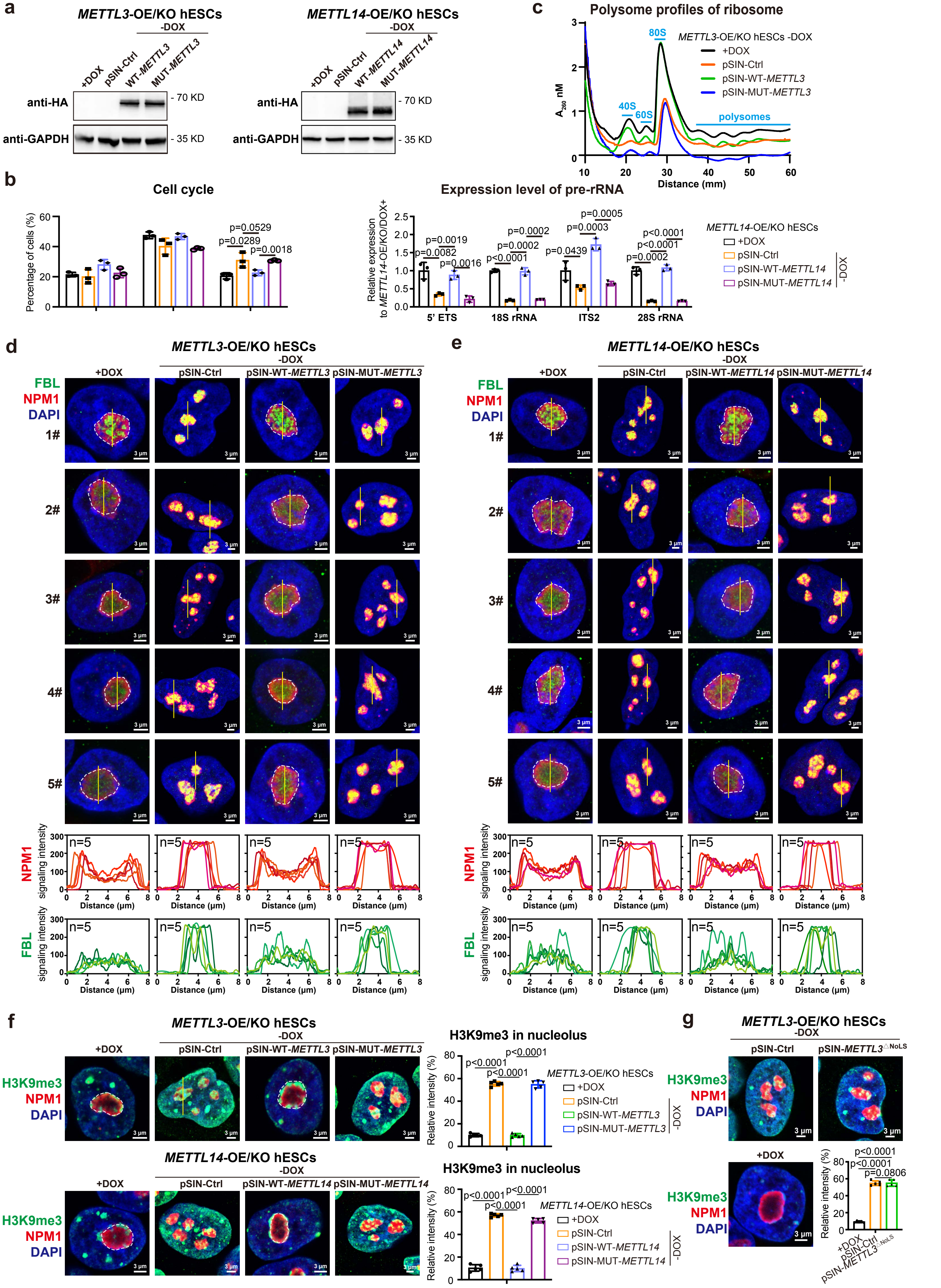

Supplementary Figure 6. METTL3/METTL14 maintain nucleoli integrity depending on their core motifs

(a) Western blot analysis of HA tagged different form of METTL3 or METTL14 in the indicated cells. **(b)** Cell cycle analysis and qRT-PCR analysis on the expression level of pre-rRNA in the indicated cell lines. The data represent mean ± SD from three independent experiments (n=3). Statistical analysis, unpaired two-tailed Student's t-test. **(c)** Polysome profiling in the indicated cell lines was analyzed using by sucrose density-gradient ultracentrifugation. (**d-e)** Five representative immunostaining analysis on FBL and NPM1 in the indicated cells. Scale bar, 3 μm. (**f-g)** Immunostaining analysis on H3K9me3 and NPM1 and quantification for H3K9me3 in nucleolus in the indicated cells. Scale bar, 3 μm. The data represent mean ± SD from five independent experiments (n=5). Statistical analysis, unpaired two-tailed Student's t-test.

Supplementary Figure 7

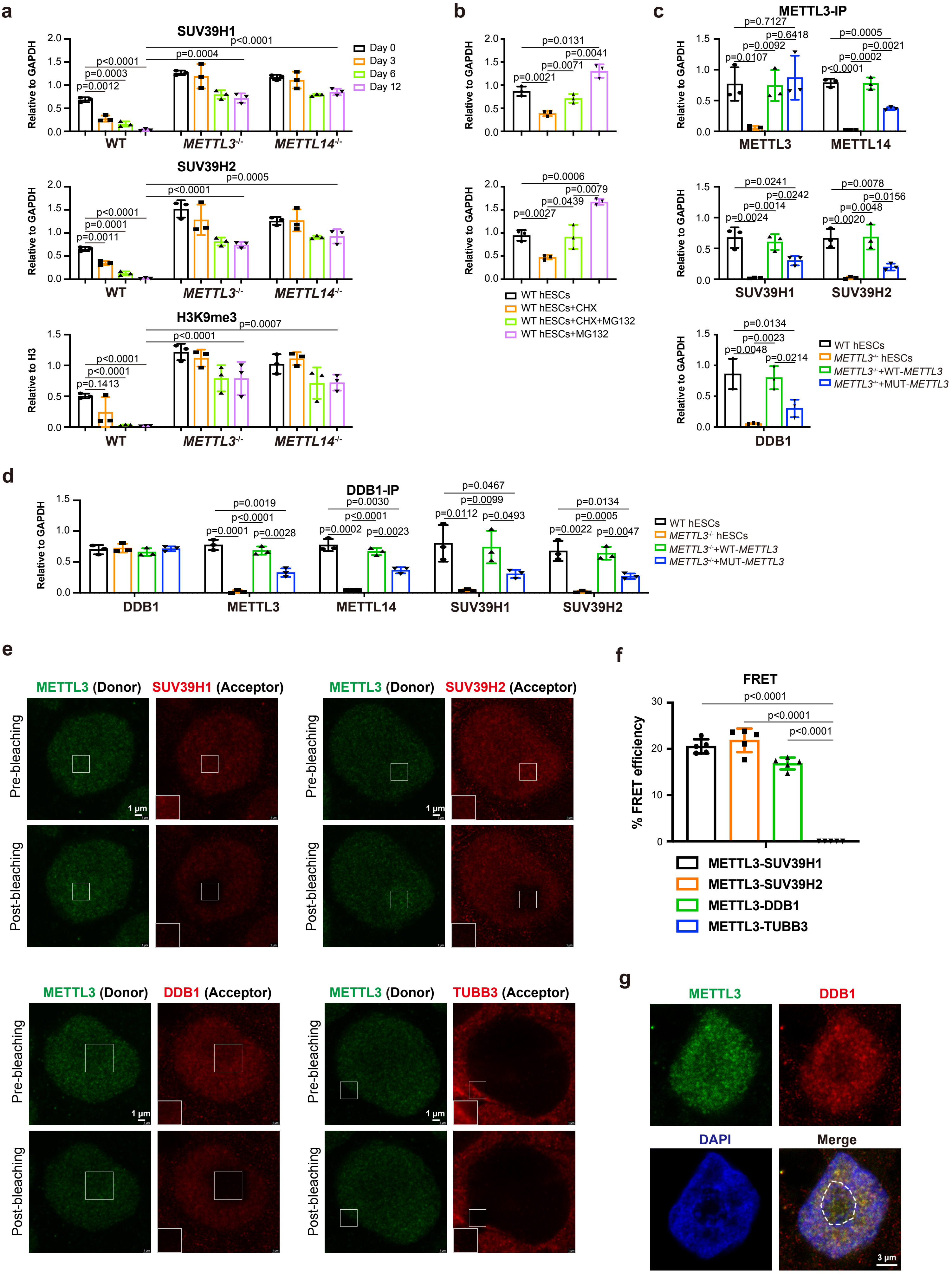

Supplementary Figure 7. METTL3/METTL14 mediate SUV39H1/H2 proteasomal degradation as an essential adaptor for CRL4 E3 ubiquitin ligase

(a) The quantification results of western blot analysis for SUV39H1, SUV39H2, and H3K9me3 in **Fig. 5b**. **(b)** The quantification results of western blot analysis for SUV39H1 and SUV39H2 in **Fig. 5c**. **(c)** The quantification results of western blot analysis for METTL3, METTL14, SUV39H1, SUV39H2, and DDB1 in METTL3 Co-IP assay in **Fig. 5e**. **(d)** The quantification results of western blot analysis for METTL3, METTL14, SUV39H1, SUV39H2, and DDB1 in DDB1 Co-IP assay in **Fig. 5e**. **(e-f)** The representative images (**e**) and quantification result (**f**) during FRET of METTL3 and SUV39H1, SUV39H2, and DDB1 in wild type (WT) hESCs, respectively. Scale bar, 1 μm. **(g)** Immunostaining analysis on METTL3 and DDB1 in wild-type (WT) hESCs. Scale bar, 3 μm. The data in **Supplementary Fig. 7a-d** represent mean ± SD from three independent experiments (n=3) and in **Supplementary Fig. 7f** represent mean ± SD from five independent experiments (n=5). Statistical analysis, unpaired two-tailed Student's t-test.

Supplementary Figure 8

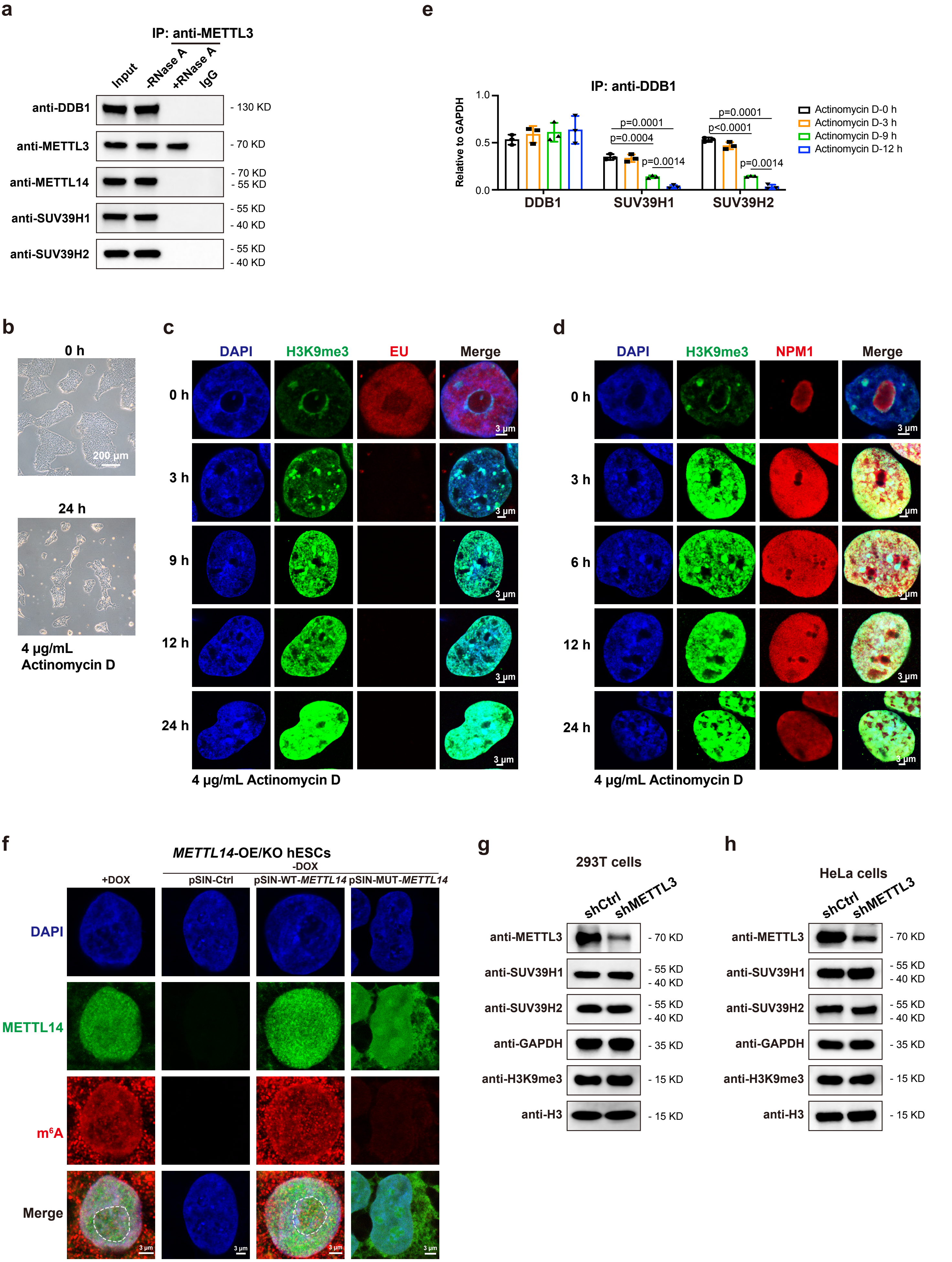

Supplementary Figure 8. METTL3/METTL14 mediate SUV39H1/H2 proteasomal degradation depend on nascent RNA

(a) METTL3 Co-IP assay and western blot analysis for DDB1, METTL14, SUV39H1 and SUV39H2 in WT hESCs upon RNase treatment. **(b)** Morphology of wild type (WT) hESCs upon 4 μg/mL Actinomycin D treatment for 24 hours. Scale bar, 200 μm. **(c)** Immunostaining analysis on H3K9me3 and EU (nascent RNA) in wild type (WT) hESCs upon 4 μg/mL Actinomycin D treatment at 3, 9, 12, 24 hours. Scale bar, 3 μm. **(d)** Immunostaining analysis on H3K9me3 and NPM1 (nucleoli) in wild type (WT) hESCs upon 4 μg/mL Actinomycin D treatment at 3, 6, 12, 24 hours. Scale bar, 3 μm. **(e)** The quantification results of western blot analysis for SUV39H1, SUV39H2, and DDB1 in DDB1 Co-IP assay in **Fig. 5f**. The data represent mean ± SD from three independent experiments (n=3). Statistical analysis, unpaired two-tailed Student's t-test. **(f)** Immunostaining results on METTL14 and m^6A in the indicated cell lines. Scale bar, 3 μm. **(g-h)** Western blot of H3K9me3 and its methyltransferases SUV39H1/2 in *METTL3*-knockdown 293T and HeLa cells.

Supplementary Table 1 List of sgRNAs and primers for gene targeting

Gene	*METTL3*		*METTL14*	
sgRNA sequencing	CAGGATCTGTAGCTAATTC		GCTTGCAGGAGATCCGGGAG	
Primers for donor DNA construction	*METTL3*-5’ arm-F	5' CACCACACCAAGCCCTGAGTCA 3'	*METTL14*-5’ arm-F	5' AAAACGACGGCCAGTGAATTCCTTGCCCACTTTTGGCCCAG 3'
	METTL3-5’ arm-R	5' TTAGGGCCACCAGAGGTGGGT 3'	*METTL14*-5’ arm-R	5' CTCGAGGTTTAAACATCGATCTATTATCACCGGCTTTTCACTATCCCGAGTAC 3'
	METTL3-3’ arm-F	5' ACCACCTCTCTGATCTGGCCTTAAC 3'	*METTL14*-3’ arm-F	5' TCGACAGATCTGTTTAAACGTTTTTCTGTCCCTTCTCTACCTGCC 3'
	METTL3-3’ arm-R	5' TGAAATTAAAATGGAAGAGGCCTGG 3'	*METTL14*-3’ arm-R	5' GATTACGCCAAGCTTGGATCGTCACTGAAGCATTCCGATTTTGAG 3'
Primers for validation	*METTL3*-F1	5' GGATCCCAGCCTAGGTTGTTGAG 3'	*METTL14*-F1	5' CGTCTAGGAAGTGAGAGGCCAAGG 3'
	METTL3-R1	5' ACCTCGCTTTACCTCAATCAACTCC 3'	*METTL14*-R1	5' CATGACACTCATCCTGGCGTGAAC 3'
	METTL3-F2	5' TATGCTATACGAAGTTATGTCGAGTACCG 3'	*METTL14*-F2	5' TATGCTATACGAAGTTATGTCGAGTACCG 3'
	METTL3-R2	5' TGTTGAGATTACAGGAATGAGCCATTG 3'	*METTL14*-R2	5' TTATGCTTTCAGTTATACAGCGGCTCC 3'

Supplementary Table 2 List of primers for qRT-PCR

qPCR		
Gene	Forward primer	Reverse primer
GAPDH	GGAGCGAGATCCCTCCAAAAT	GGCTGTTGTCATACTTCTCATGG
OCT4	CCTCACTTCACTGCACTGTA	CAGGTTTTCTTTCCCTAGCT
NANOG	TGAACCTCAGCTACAAACAG	TGGTGGTAGGAAGAGTAAAG
SOX2	CCCAGCAGACTTCACATGT	CCTCCCATTTCCCTCGTTTT
PAX6	ATGTGTGAGTAAAATTCTGGGCA	GCTTACAACTTCTGGAGTCGCTA
SOX1	AATTTTATTTTCGGCGTTGC	TGGGCTCTGTCTCTTAAATTTGT
T	TATGAGCCTCGAATCCACATAGT	CCTCGTTCTGATAAGCAGTCAC
CXCR4	ACGCCACCAACAGTCAGAG	AGTCGGGAATAGTCAGCAGGA
SOX17	CGCACGGAATTTGAACAGTA	GGATCAGGGACCTGTCACAC
FOXA2	ACTACCCCGGCTACGGTTC	AGGCCCGTTTTGTTCGTGA
METTL3	TTGTCTCCAACCTTCCGTAGT	CCAGATCAGAGAGGTGGTGTAG
METTL14	AAGTACTCGGGATAGTGAAAAGCCG	CATCTTTGCTATTTAACACGGCACC
5'ETS	CTGTCGCTGGAGAGGTTGGG	GACGAGAACGCCTGACACGC
ITS2	GCGAAGACGGAGAGGGAAAGAG	ACCACCACACCGCACGCAAC
18S rRNA	CTGGATACCGCAGCTAGGAA	GAATTTCACCTCTAGCGGCG
5.8S rRNA	GTGGATCACTCGGCTCGTG	GCAAGTGCGTTCGAAGTGTC
28S rRNA	AGTAACGGCGAGTGAACAGG	GATCAGAAGGACTTGGGCCC
SUV39H1	CCTGCCCTCGGTATCTCTAAG	ATATCCACGCCATTTCACCAG
SUV39H2	TCTATGACAACAAGGGAATCACG	GAGACACATTGCCGTATCGAG

Supplementary Table 3 List of antibodies used in this study

Name of Antibody (clone name)	Company (Cat. No.)	lot number	Dilution Factor
Rabbit anti-METTL3 (E3F2A)	Cell Signaling Technology (86132)	2	1:1000 (WB); 1:1000 (IF); 1:150 (co-IP)
Rabbit anti-METTL14 (Ag14325)	proteintech (26158-1-AP)	00094149	1:1000 (WB); 1:1000 (IF)
HRP-conjugated Monoclonal Mouse Anti-GAPDH (1E6D9)	Proteintech (HRP-60004)	21010938	1:10000
Mouse anti-P53 (DO-7)	Cell Signaling Technology (48818)	4	1:1000 (WB)
Mouse anti-OCT3/4 (C-10)	Santa Cruz Biotechnology (SC-5279)	G1423	1:100 (IF and FACS)
Mouse anti-NPM1 (FC-61991)	invitrogen (325200)	YE373722	1:1000 (IF)
Goat anti Rabbit IgG HRP	KangChen Bio-tech (KC-RB-035)	1803	1:1000
Goat anti Mouse IgG HRP	KangChen Bio-tech (KC-MM-035)	1807	1:1000
Goat anti Goat IgG HRP	Proteintech (SA00001-4)	00078078	1:2000
Goat anti-Mouse IgG-488	Invitrogen (A11001)	2610355	1:500
Goat anti-Mouse IgG-568	Invitrogen (A11004)	2198584	1:1000
Goat anti-Rabbit IgG-488	Invitrogen (A11008)	2747438	1:1000
Donkey anti-Goat IgG-647	Invitrogen (A21447)	2297623	1:1000
Mouse anti-HA (HA-7)	Sigma (H3663)	038M4810V	1:1000 (WB)
Rabbit anti-NANOG (D73G4)	Cell Signaling Technology (4903T)	3	1:1000 (IF)
Mouse anti-SOX2 (245610)	R&D (mab2018)	KGQ0317081	1:1000 (IF)
Mouse anti-SOX17 (245013)	R&D (mab1924)	KGA1022032	1:1000 (IF)
Rabbit anti-NESTIN	Millipore (ABD69)	3537114	1:1000 (IF)
Rabbit anti-CALPONIN (EP798Y)	Abcam (ab46794)	GR3234463-2	1:1000 (IF)
Rabbit anti-PAX6 (Poly19013)	Biolegend (PRB-278P)	B277104	1:1000 (IF)
Mouse anti-UBF (F-9)	Santacruz (sc-13125)	J2422	1:100 (IF)
Rabbit anti-FBL (C13C3)	Cell Signaling Technology (2639)	4	1:200 (IF)
Rabbit anti-H3K9me3 (ab1773)	Abcam (ab8898)	1063771-1	1:1000 (WB); 1:500 (IF); 1:50 (CUT&TAG)
Mouse anti-SUV39H1 (44.1)	Novus (NB120-12405)	151802	1:200 (IF); 1:1000 (WB)
Goat anti-SUV39H2 (CKCGAVTCRGYLN)	Novus (NB100-1140)	G2	1:200 (IF); 1:1000 (WB)
Mouse anti-m^6A (212B11)	Synaptic Systems (202111)	1-21	1:200 (IF)
Mouse anti-H3 (ab12149)	Abcam (Ab1791)	1015880-5	1:5000 (WB)
Mouse anti-FLAG (M2)	Sigma (F1804)	SLCM4081	1:1000 (WB); 1:150 (co-IP)
Rabbit anti-DDB1 (EPR6089)	Abcam (ab109027)	1018022-9	1:5000 (WB); 1:150 (co-IP)
Rabbit anti-MYC (Ag9409)	Proteintech (16286-1-AP)	00132370	1:5000 (WB)
Rabbit anti-SETDB1 (Ag1725)	Proteintech (11231-1-AP)	00073102	1:1000 (WB)

Supplementary Table 4 List of primer sequences for over-expression

Primers for *METTL3*-WT		Primers for *METTL14*-WT	
METTL3-WT-F	5' CTAGCTAGCATGTCGGACACGTGGAGCTCTATCC 3'	*METTL14*-WT-F	5' CCGGAATTCATGGATAGCCGCTTGCAGGAG 3'
METTL3-WT-R	5' CCATCGATTAAATTCTTAGGTTTAGAGATGATACCATCTGGG 3'	*METTL14*-WT-R	5' CGCGGATCCTCGAGGTGGAAAGCCACCTCTG 3'
Primers for *METTL3*-Mut		Primers for *METTL14*-Mut	
METTL3-Mut-F1	5' ATTACGCTGAATTCGCTAGCATGTCGGACACG 3'	*METTL14*-Mut-F1	5' CCAGATTACGCTGAATTCATGGATAGCCGCTTG 3'
METTL3-Mut-R1	5' TGAATATCTGCGGGTGGTGCAGCCATCACAACTG 3'	*METTL14*-Mut-R1	5' CTGTAATATTCTTCTGCAGGGGGTGCCAGAAGAATCACATCAAATT 3'
METTL3-Mut-F2	5' TGCACCACCCGCAGATATTCACATGGAACTGCC 3'	*METTL14*-Mut-F2	5' CTTCTGGCACCCCCTGCAGAAGAATATTACAGAGAAACTGG 3'
METTL3-Mut-R2	5' CTTTCTCGAGGCCATCGATCTATAAATTCTTAGGT 3'	*METTL14*-Mut-R2	5' CCTAGATGCATGCGGATCCTTATCGAGGTGGAAAG 3'

Primers for GFP-NPM1		Primers for mCherry-H2B	
NPM1-F	5' ATGGAAGATTCGATGGACATGGAC 3'	*H2B*-F	5' ATGCCTGAACCCTCTAAGTCTGCTC 3'
NPM1-R	5'GCTCCTCGCCCTTGCTCACCATAAGAGACTTCCTCCACTGCCA GAGA 3'	*H2B*-R	5'CTCCTCGCCCTTGCTCACCATTTTAGAGCTAGTGTACTTGGTAACTG CCTT 3'
GFP-F	5' ATGGTGAGCAAGGGCGAGGAGC 3'	mCherry-F	5' ATGGTGAGCAAGGGCGAGGAGG 3'
GFP-R	5' TTACTTGTACAGCTCGTCCATGCCG 3'	mCherry-R	5' TTACTTGTAGAGCTCGTCCATGCC 3'

Primers for SUV39H1-OE		Primers for SUV39H2-OE	
SUV39H1-F	5' ATGGCGGAAAATTTAAAAGGCTGCAGC 3'	*SUV39H2*-F	5' ATGGAATATTATCTTGTAAAATGGAAAGGATGGCC 3'
SUV39H1-R	5' CTAGAAGAGGTATTTGCGGCAGGACTC 3'	*SUV39H2*-R	5'TCAGTTGAGGTAACCTCTGCAAGTCAC 3'

Primers for ubiquitin			
ubiquitin-F	5' ATGCAGATCTTCGTGAAGACCCTGA 3'		
ubiquitin-R	5' TTACCCACCTCTGAGACGGAGCA 3'		

Uncropped gels and blots

Supplementary Fig. 2c

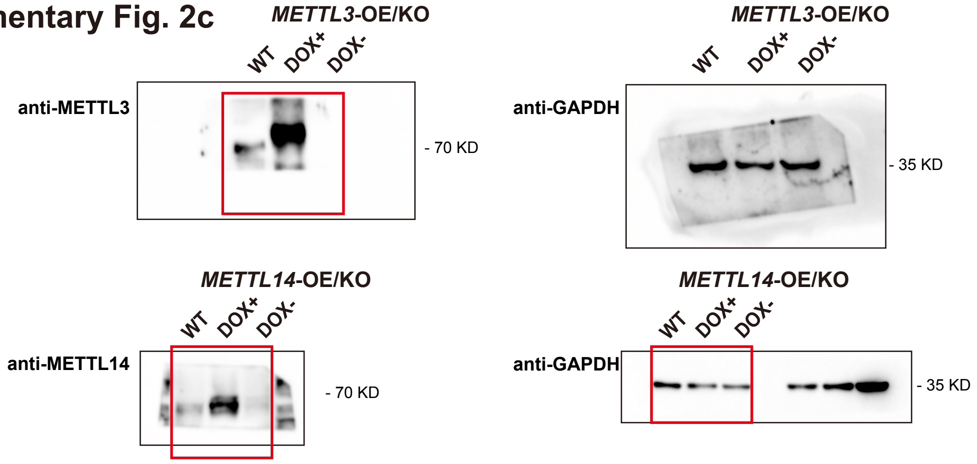

Supplementary Fig. 3e

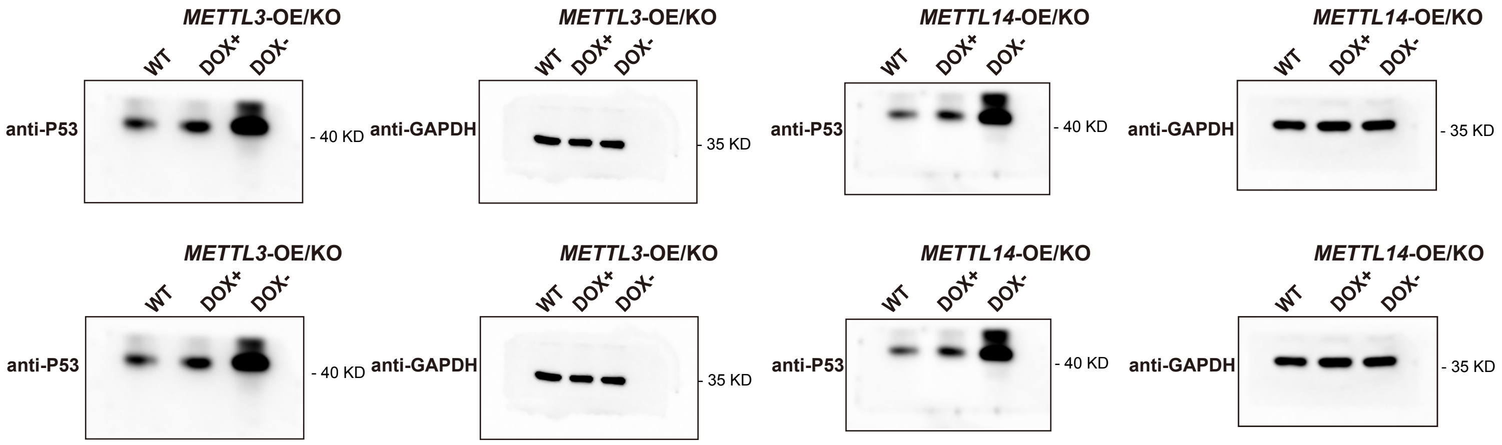

Supplementary Fig. 5b

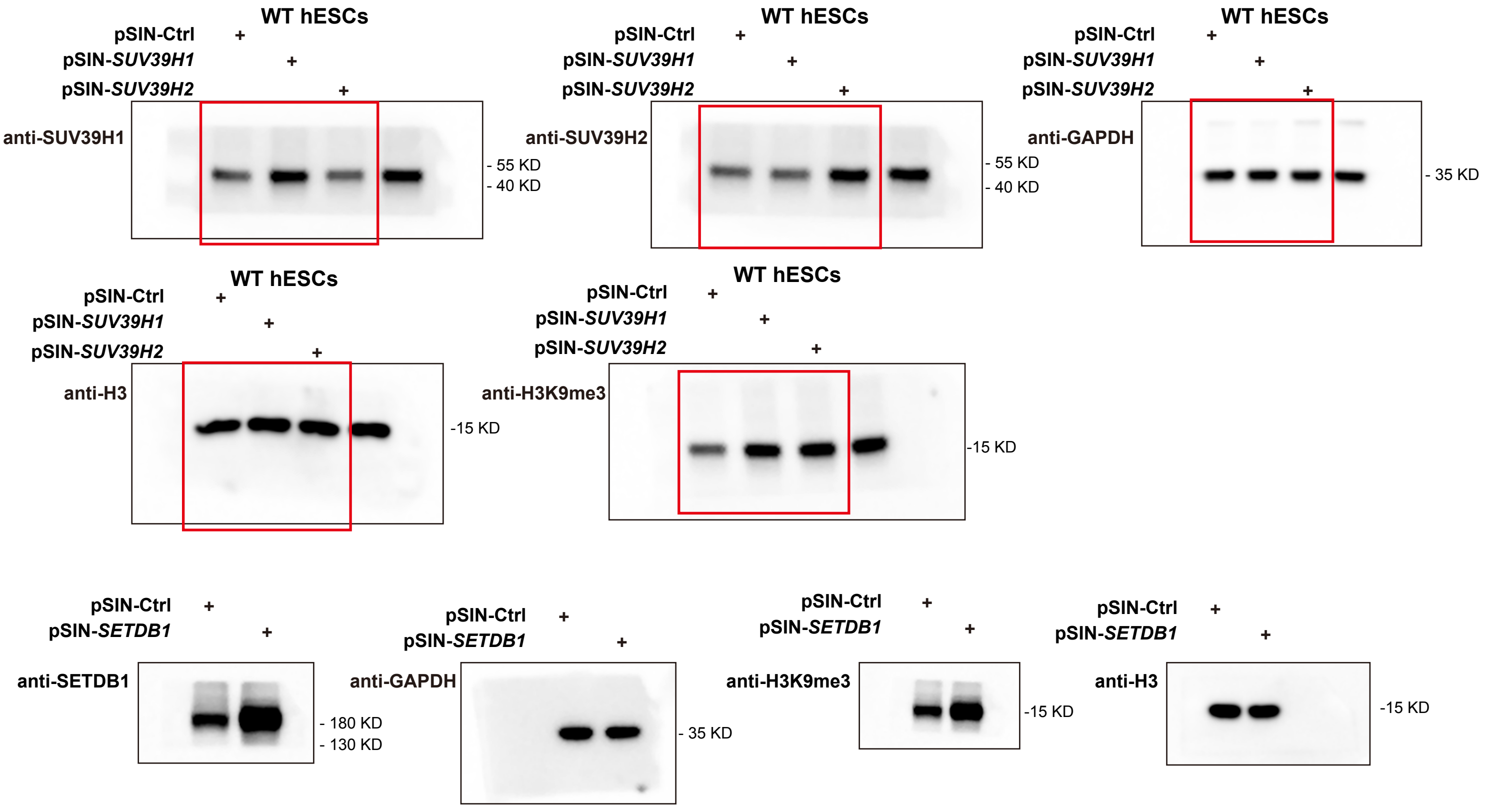

Supplementary Fig. 6a

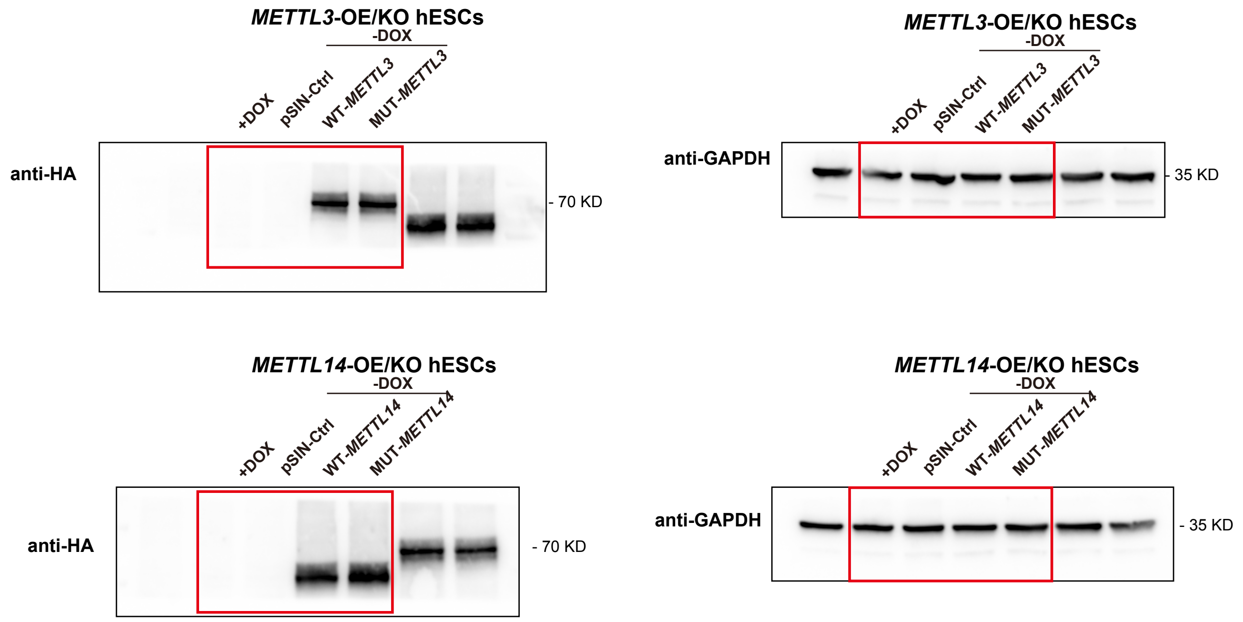

Supplementary Fig. 8a

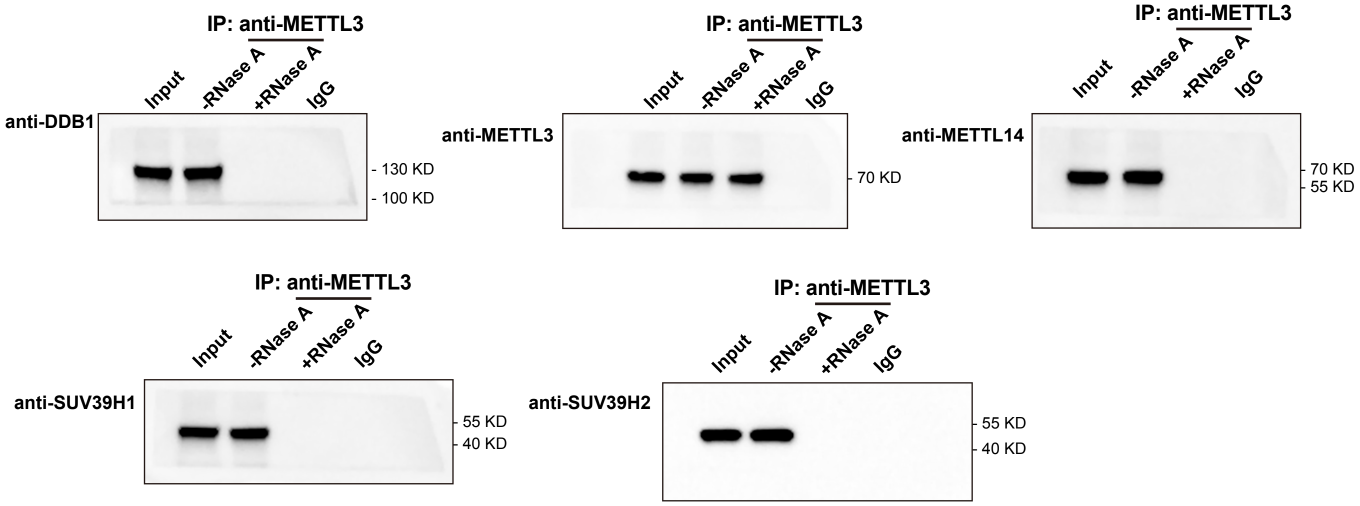

Supplementary Fig. 8g

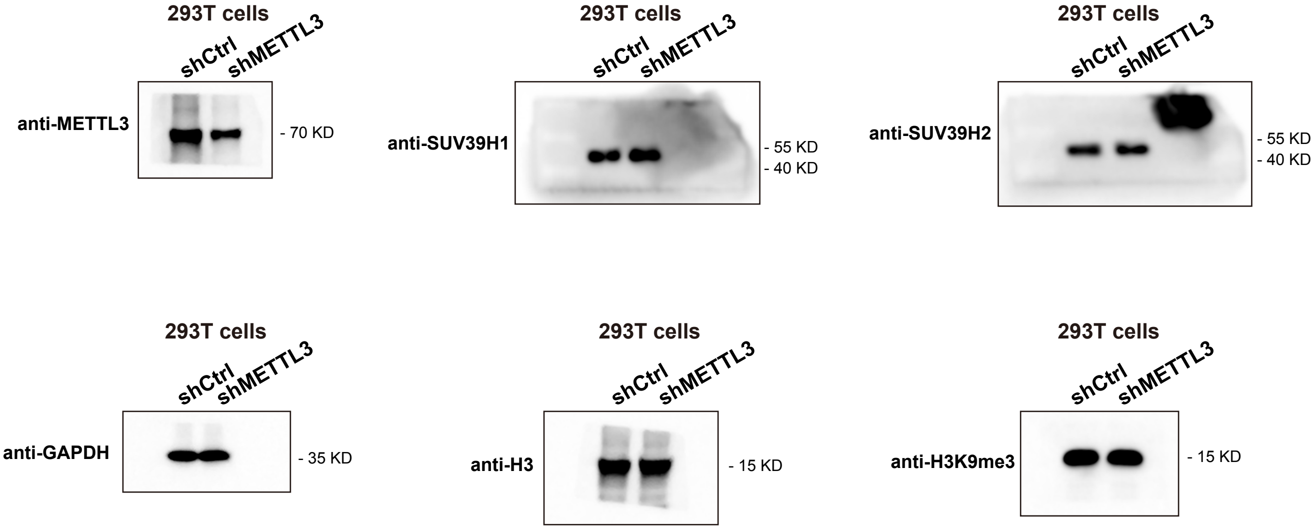

Supplementary Fig. 8h

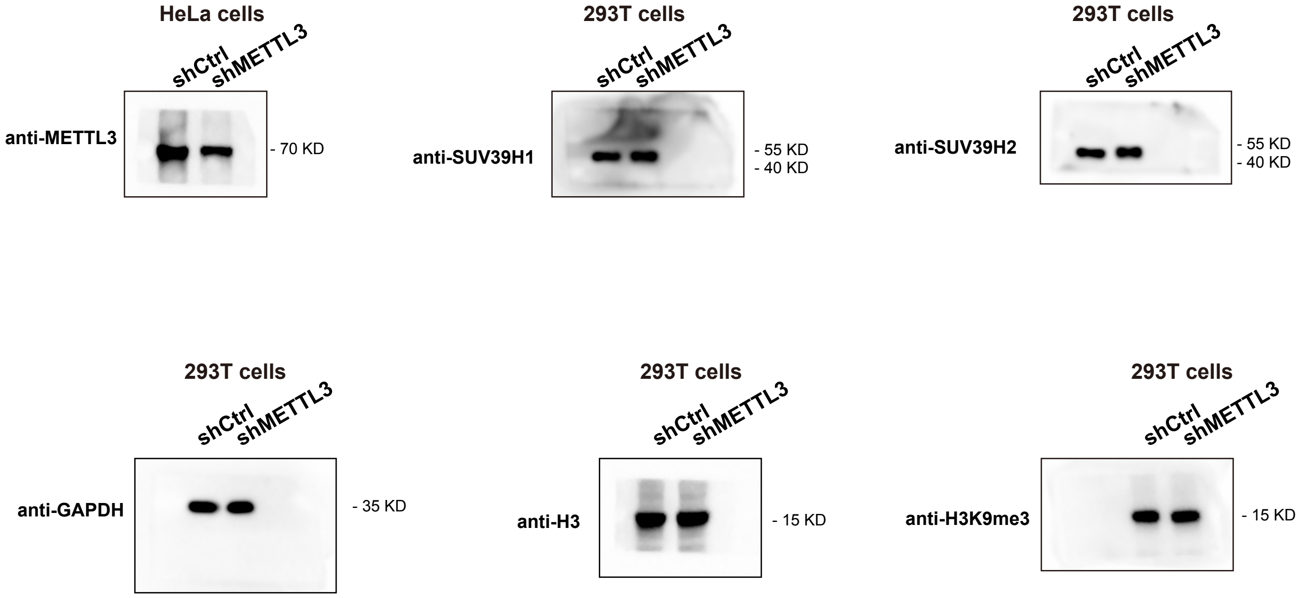

Supplement: Supplementary file 1 — Supplementary Information [file 41467_2024_51742_MOESM1_ESM.pdf]
