# Supplementary material for: METTL3/METTL14 maintain human nucleoli integrity by mediating SUV39H1/H2 degradation
Source: Nat Commun. 2024 Aug 21;15:7186. doi: 10.1038/s41467-024-51742-7 (PMC11339338; doi:10.1038/s41467-024-51742-7)
Supplement: Supplementary file 3 — Reporting Summary [file 41467_2024_51742_MOESM3_ESM.pdf]

nature portfolio

Corresponding author(s): Guangjin Pan

Last updated by author(s): Jul 27, 2024

Reporting Summary

Nature Portfolio wishes to improve the reproducibility of the work that we publish. This form provides structure for consistency and transparency in reporting. For further information on Nature Portfolio policies, see our Editorial Policies and the Editorial Policy Checklist.

Statistics

For all statistical analyses, confirm that the following items are present in the figure legend, table legend, main text, or Methods section.

n/a	Confirmed	
☐	☒	The exact sample size (*n*) for each experimental group/condition, given as a discrete number and unit of measurement
☐	☒	A statement on whether measurements were taken from distinct samples or whether the same sample was measured repeatedly
☐	☒	The statistical test(s) used AND whether they are one- or two-sided<br>*Only common tests should be described solely by name; describe more complex techniques in the Methods section.*
☐	☒	A description of all covariates tested
☒	☐	A description of any assumptions or corrections, such as tests of normality and adjustment for multiple comparisons
☐	☒	A full description of the statistical parameters including central tendency (e.g. means) or other basic estimates (e.g. regression coefficient) AND variation (e.g. standard deviation) or associated estimates of uncertainty (e.g. confidence intervals)
☒	☐	For null hypothesis testing, the test statistic (e.g. *F*, *t*, *r*) with confidence intervals, effect sizes, degrees of freedom and *P* value noted<br>*Give P values as exact values whenever suitable.*
☒	☐	For Bayesian analysis, information on the choice of priors and Markov chain Monte Carlo settings
☒	☐	For hierarchical and complex designs, identification of the appropriate level for tests and full reporting of outcomes
☒	☐	Estimates of effect sizes (e.g. Cohen's *d*, Pearson's *r*), indicating how they were calculated

Our web collection on statistics for biologists contains articles on many of the points above.

Software and code

Policy information about availability of computer code

Data collection	In general, results were presented as mean ± SD calculated using Microsoft Excel and GraphPad Prism at least three biological repeats.<br>Significance level between samples was determined using unpaired two-tailed Student’s t-tests. P value <0.05 was considered statistically significant in the figures. No samples were excluded for any analysis.<br>For FACS, cells were collected on a Beckman CytoFlex.<br>For IF, images were captured by Zeiss LSM 800 microscope and SP8-STED.<br>For RNA-seq, CUT&TAG, our data collection processes were described in methods and materials section of this manuscript.
Data analysis	We used Microsoft Excel and GraphPad Prism to analyze these data.<br>For FACS, these data were analyzed using flowjo.<br>For IF, fluorescence intensity and fluorescence localization analysis were done by Image J.<br>For RNA-seq and CUT&TAG, our data analysis processes were described in methods and materials section of this manuscript.

For manuscripts utilizing custom algorithms or software that are central to the research but not yet described in published literature, software must be made available to editors and reviewers. We strongly encourage code deposition in a community repository (e.g. GitHub). See the Nature Portfolio guidelines for submitting code & software for further information.

Data

Policy information about availability of data

All manuscripts must include a data availability statement. This statement should provide the following information, where applicable:

- Accession codes, unique identifiers, or web links for publicly available datasets
- A description of any restrictions on data availability
- For clinical datasets or third party data, please ensure that the statement adheres to our policy

The raw data of RNA sequencing and CUT&TAG sequencing has been deposited in the Genome Sequence Archive under the accession code HRA002951 and HRA007207. qRT-PCR data, original western blots, the quantification results of FACS, corresponding western blot, and fluorescence strength have also been deposited in Figshare (https://doi.org/10.6084/m9.figshare.25623738). Source data are provided with this paper. The datasets in this study are available from the corresponding author upon reasonable request.

Research involving human participants, their data, or biological material

Policy information about studies with human participants or human data. See also policy information about sex, gender (identity/presentation), and sexual orientation and race, ethnicity and racism.

Reporting on sex and gender	In this study, human embryonic stem cell line H1 and its knockout cells were used. No sex and gender were used to determine the results.
Reporting on race, ethnicity, or other socially relevant groupings	In this study, human embryonic stem cell line H1 and its knockout cells were used. No race, ethnicity, or other socially relevant groupings were used.
Population characteristics	In this study, human embryonic stem cell line H1 and its knockout cells were used. No population characteristics were used.
Recruitment	In this study, human embryonic stem cell line H1 and its knockout cells were used. No samples were recruited.
Ethics oversight	In this study, human embryonic stem cell line H1 and its knockout cells were used according to ISSCR and institutional guidelines.

Note that full information on the approval of the study protocol must also be provided in the manuscript.

Field-specific reporting

Please select the one below that is the best fit for your research. If you are not sure, read the appropriate sections before making your selection.

☒ Life sciences ☐ Behavioural & social sciences ☐ Ecological, evolutionary & environmental sciences

For a reference copy of the document with all sections, see nature.com/documents/nr-reporting-summary-flat.pdf

Life sciences study design

All studies must disclose on these points even when the disclosure is negative.

Sample size	In this study, human embryonic stem cells were used. No statistical methods were used to determine sample size. We chose the sample size based on literatures in the field.
Data exclusions	No exclusions.
Replication	Phenotypes observed are robust and were reliably reproduced at least three biological repeats. RNA-seq data were analyzed with two repeats. CUT&TAG data were performed one time.
Randomization	In this study, we analyzed cell morphology, qRT-PCR data, Western blot data, FACS, RNA-seq data, and CUT&TAG data with cell populations. We analyzed immuno-staining data with at least three random selected pictures.
Blinding	No blinding was used in this study.

Reporting for specific materials, systems and methods

We require information from authors about some types of materials, experimental systems and methods used in many studies. Here, indicate whether each material, system or method listed is relevant to your study. If you are not sure if a list item applies to your research, read the appropriate section before selecting a response.

Materials & experimental systems

n/a	Involved in the study
☐	☒ Antibodies
☐	☒ Eukaryotic cell lines
☒	☐ Palaeontology and archaeology
☐	☒ Animals and other organisms
☒	☐ Clinical data
☒	☐ Dual use research of concern
☒	☐ Plants

Methods

n/a	Involved in the study
☐	☒ ChIP-seq
☐	☒ Flow cytometry
☒	☐ MRI-based neuroimaging

Antibodies

Antibodies used

The detail information of antibodies used in this study was listed in Supplementary Table 3. Please see Supplementary Table 3.

Validation

Rabbit anti-HIST3H3 (H3), Abcam, Cat. ab1791; Clone name: ab12149; Lot number: 1015880-5; 1:5,000; western blot validation in Fig. 3a and peer-reviewed citations at https://www.abcam.cn/products/primary-antibodies/histone-h3-antibody-nuclear-marker-and-chip-grade-ab1791.html.
HRP-conjugated Monoclonal Mouse Anti-GAPDH, Proteintech, Cat. HRP-60004; Clone name: 1E6D9; Lot number: 21010938; 1:1,0000; western blot validation in Fig. 1a and peer-reviewed citations at https://www.ptglab.com/products/GAPDH-Antibody-HRP-60004.htm.
Goat anti Rabbit IgG HRP, KangChen Bio-tech, Cat. KC-RB-035; Lot number: 1803; 1:4,000; western blot validation in Fig. 1a and peer-reviewed citations at http://www.aksomics.com/products/secondary-antibody.html.
mouse anti-OCT-3/4, Santa Cruz Biotechnology, Cat. sc-5279; Clone name: C-10; Lot number: G1423; 1:100; FACS validation in Fig. S2h and peer-reviewed citations at https://www.scbt.com/scbt/product/oct-3-4-antibody-c-10?requestFrom=search#thumbcarousel.
Goat anti-Mouse IgG (H+L) Cross-Adsorbed Secondary Antibody, Alexa Fluor™ 488, Invitrogen, Cat. A11001; Lot number: 2610355; 1:500; FACS validation in Fig. S1f and peer-reviewed citations at https://www.thermofisher.cn/cn/en/antibody/product/Goat-anti-Mouse-IgG-H-L-Cross-Adsorbed-Secondary-Antibody-Polyclonal/A-11001.
Goat anti-Rabbit IgG (H+L) Cross-Adsorbed Secondary Antibody, Alexa Fluor 488, Invitrogen, Cat. A-11008; Lot number: 2747438; 1:1000, immune-staining validation in Fig. 2b and peer- reviewed citations at https://www.thermofisher.com/antibody/product/Goat-anti-Rabbit-IgG-H-L-Cross-Adsorbed-Secondary-Antibody-Polyclonal/A-11008.
Goat anti-Mouse IgG (H+L) Cross-Adsorbed Secondary Antibody, Alexa Fluor 568, Invitrogen, Cat. A-11004; Lot number: 2198584; 1:1000; immune-staining validation in Fig. 2b and peer-reviewed citations at https://www.thermofisher.com/antibody/product/Goat-anti-Mouse-IgG-H-L-Cross-Adsorbed-Secondary-Antibody-Polyclonal/A-11004.
Donkey anti-Goat IgG (H+L) Cross-Adsorbed Secondary Antibody, Alexa Fluor™ 647, Invitrogen, Cat. A21447; Lot number: 2297623; 1:1000; immune-staining validation in Fig. 3e and peer-reviewed citations at https://www.thermofisher.cn/cn/en/antibody/product/Donkey-anti-Goat-IgG-H-L-Cross-Adsorbed-Secondary-Antibody-Polyclonal/A-21447.
Mouse anti-SOX2, R&D system, Cat. MAB2018; Clone name: 245610; Lot number: KGQ0317081; 1:1000; immune-staining validation in Fig. S1f and peer-reviewed citations at https://www.rndsystems.com/cn/products/human-mouse-rat-sox2-antibody-245610_mab2018.
Rabbit anti-PAX6, BioLegend, Cat.901301; Clone name: Poly19013; Lot number: B277104; 1:1000; western blot validation in Fig. S1f and peer-reviewed citations at https://www.biolegend.com/en-us/products/purified-anti-pax-6-antibody-11511.
Mouse anti-FLAG, Sigma-Aldrich, Cat. F1804; Clone name: M2; Lot number: SLCM4081; 1:1000; western blot validation in Fig.5d and peer-reviewed citations at https://www.sigmaaldrich.com/catalog/product/sigma/f1804?lang=zh&region=CN.
Rabbit anti-METTL3, Cell Signaling Technology, Cat. 86132;Clone name: E3F2A; Lot number: 2; 1:1000 (WB); 1:1000 (IF); 1:150 (co-IP); western blot and immune-staining validation in Fig. 1a and 4d and peer-reviewed citations at https://www.cellsignal.cn/products/primary-antibodies/mettl3-e3f2a-rabbit-mab/86132.
Rabbit anti-METTL14, Proteintech, Cat. 26158-1-AP; Clone name: Ag14325; Lot number: 00094149; 1:1000 (WB); 1:1000 (IF); western blot and immune-staining validation in Fig. 1a and 4d and peer-reviewed citations at https://www.ptgcn.com/products/METTL14-Antibody-26158-1-AP.htm.
Mouse anti-P53,Cell Signaling Technology, Cat. 48818, Clone name: DO-7; Lot number: 4; 1:1000; western blot validationin Fig. 1i and peer-reviewed citations at https://www.cellsignal.cn/products/primary-antibodies/p53-do-7-mouse-mab/48818.
Mouse anti-NPM1, invitrogen, Cat. 325200; Clone name: FC-61991; Lot number: YE373722; 1:1000 (IF); immune-staining validation in Fig. 1e and peer-reviewed citations at https://www.thermofisher.cn/cn/en/antibody/product/NPM1-Antibody-clone-FC-61991-Monoclonal/32-5200.
Goat anti Mouse IgG HRP, KangChen Bio-tech, Cat. KC-MM-035; Lot number: 1807; 1:1000; western blot validation in Fig. 1i and peer-reviewed citations at http://www.aksomics.com/products/secondary-antibody.html.
Goat anti Goat IgG HRP, Proteintech, Cat. SA00001-4; Lot number: 00078078; 1:2000; western blot validation in Fig. 3c and peer-reviewed citations at https://www.ptgcn.com/products/HRP-conjugated-Affinipure-Rabbit-Anti-Goat-IgG-H-L-secondary-antibody.htm.
Mouse anti-HA, Sigma, Cat. H3663; Clone name: HA-7; Lot number: 038M4810V; 1:1000; western blot in Fig. 5d and peer-reviewed citations at https://www.sigmaaldrich.cn/CN/zh/product/sigma/h3663.
Rabbit anti-NANOG, Cell Signaling Technology, Cat. 4903T; Clone name: D73G4; Lot number: 3; 1:1000 (IF); immune-staining validation in Fig. S1f and peer-reviewed citations at https://www.cellsignal.cn/products/primary-antibodies/nanog-d73g4-xp-rabbit-mab/4903.
Mouse anti-SOX17, R&D, Cat. mab1924; Clone name: 245013; Lot number: KGA1022032; 1:1000 (IF); immune-staining validation in Fig. S1f and peer-reviewed citations at https://www.rndsystems.com/cn/products/human-sox17-antibody-245013_mab1924.
Rabbit anti-NESTIN, Millipore, Cat. ABD69; Lot number: 3537114; 1:1000 (IF); immune-staining validation in Fig. S1f and peer-reviewed citations at https://www.sigmaaldrich.cn/CN/zh/product/mm/abd69.
Rabbit anti-CALPONIN, Abcam, Cat. ab46794; Clone name: EP798Y; Lot number: GR3234463-2; 1:1000 (IF); immune-staining validation in Fig. S1f and peer-reviewed citations at https://www.abcam.com/products/primary-antibodies/calponin-1-antibody-

ep798y-ab46794.html.
Mouse anti-UBF, Santacruz, Cat. sc-13125; Clone name: F-9; Lot number: J2422; 1:100 (IF); immune-staining validation in Fig. 2c and peer-reviewed citations at https://www.scbt.com/zh/p/ubf-antibody-f-9.
Rabbit anti-FBL, Cell Signaling Technology, Cat. 2639; Clone name: C13C3; Lot number: 4; 1:200 (IF); immune-staining validation in Fig. 2b and peer-reviewed citations at https://www.cellsignal.cn/products/primary-antibodies/fibrillarin-c13c3-rabbit-mab/2639.
Rabbit anti-H3K9me3, Abcam, Cat. ab8898; Clone name: ab1773; Lot number: 1063771-1; 1:1000 (WB); 1:500 (IF); 1:50 (CUT&TAG); western blot, immune-staining, and CUT&TAG validation in Fig. 3a. 3b. S5f and peer-reviewed citations at https://www.abcam.com/products/primary-antibodies/histone-h3-tri-methyl-k9-antibody-chip-grade-ab8898.html.
Mouse anti-SUV39H1, Novus, Cat. NB120-12405; Clone name: 44.1; Lot number: 151802; 1:200 (IF); 1:1000 (WB); western blot, immune-staining validation in Fig.3c and 3d and peer-reviewed citations at https://www.novusbio.com/products/kmt1a-suv39h1-antibody-441_nb120-12405.
Goat anti-SUV39H2, Novus, Cat. NB100-1140; Immunogen: CKCGAVTCRGYLN; Lot number: G2; 1:200 (IF); 1:1000 (WB); western blot, immune-staining validation in Fig. 3c and 3e and peer-reviewed citations at https://www.novusbio.com/products/suv39h2-antibody_nb100-1140.
Rabbit anti-DDB1, Abcam, Cat. ab109027; Clone name: EPR6089;Lot number: 1018022-9; 1:5000 (WB); 1:150 (co-IP); western blot, immune-staining validation in Fig.5d and S7e and peer-reviewed citations at https://www.abcam.com/products/primary-antibodies/ddb1-antibody-epr6089-ab109027.html.
Rabbit anti-MYC, Proteintech, Cat. 16286-1-AP; Clone name: Ag9409; Lot number: 00132370; 1:5000 (WB); western blot validation in Fig. 5d and peer-reviewed citations at https://www.ptgcn.com/products/MYC-tag-Antibody-16286-1-AP.htm.
Rabbit anti-SETDB1, Proteintech, Cat. 11231-1-AP; Clone name: Ag1725; Lot number: 00073102; 1:1000 (WB); western blot validation in Fig. S5b and peer-reviewed citations at https://www.ptgcn.com/products/SETDB1-Antibody-11231-1-AP.htm.

Eukaryotic cell lines

Policy information about cell lines and Sex and Gender in Research

Cell line source(s)	H1 hESCs were purchased from Wi Cell (hPSCReg ID: WAe001-A).
Authentication	These cell lines have been authenticated by karyotyping.
Mycoplasma contamination	We have tested these cell lines for mycoplasma contamination. We found that these cell lines are not contamination with mycoplasma.
Commonly misidentified lines (See ICLAC register)	N/A

Animals and other research organisms

Policy information about studies involving animals; ARRIVE guidelines recommended for reporting animal research, and Sex and Gender in Research

Laboratory animals	Mouse
Wild animals	In the teratoma formation experiment, both male and female immuno-deficient NOD-SCID mice at the age of about 4 weeks were used.
Reporting on sex	In the teratoma formation experiment, both male and female immuno-deficient NOD-SCID mice at the age of about 4 weeks were used.
Field-collected samples	We injected these cells subcutaneously into immuno-deficient NOD-SCID mice at the age of about 4 weeks without DOX treatment. 8 weeks later, these teratomas were analyzed and fixed in 4% paraformaldehyde. Then these teratomas were stained with hematoxylin/eosin (H&E).
Ethics oversight	The experiments involving animal research for teratoma formation had been reviewed and approved by IACUC at GIBH (NO. 2010012).

Note that full information on the approval of the study protocol must also be provided in the manuscript.

Plants

Seed stocks	N/A
Novel plant genotypes	N/A
Authentication	N/A

ChIP-seq

Data deposition

☒ Confirm that both raw and final processed data have been deposited in a public database such as GEO.

☒ Confirm that you have deposited or provided access to graph files (e.g. BED files) for the called peaks.

Data access links<br>*May remain private before publication.*	The raw data of CUT&TAG sequencing has been deposited in the Genome Sequence Archive under the accession code HRA007207 (https://ngdc.cncb.ac.cn/gsa-human/s/369n4vu8)
Files in database submission	HRR1729042_f1.fq.gz<br>HRR1729042_r2.fq.gz<br>HRR1729043_f1.fq.gz<br>HRR1729043_r2.fq.gz<br>HRR1729044_f1.fq.gz<br>HRR1729044_f1.fq.gz<br>HRR1729044_r2.fq.gz<br>HRR1729045_f1.fq.gz<br>HRR1729045_r2.fq.gz
Genome browser session<br>(e.g. UCSC)	N/A

Methodology

Replicates	CUT&TAG data were performed one time.
Sequencing depth	WT-H3K9me3 (HRR1729043), 5000000 reads, 4537847 (90.76%) overall alignment rate.<br>WT-nagetive control (HRR1729042), 110434 reads, 70133 (63.51%) overall alignment rate.<br>METTL3-KO-H3K9me3 (HRR1729045), 5000000 reads, 4515413 (90.31%) overall alignment rate.<br>METTL3-KO-nagetive control (HRR1729044), 319876 reads, 231162 (72.27%) overall alignment rate.
Antibodies	In this study, we used H3K9me3 antibody to do CUT&TAG-seq assay. This antibody was purchased from Abcam. Catalog number is ab8898. Immunogen is synthetic human histone H3 (tri methyl K9) peptide (ab1773). The detail information of this antibody is available as https://www.abcam.cn/products/primary-antibodies/histone-h3-tri-methyl-k9-antibody-chip-grade-ab8898.html.
Peak calling parameters	MACS2 (v2.2.6) was used for peak calling of H3K9me3 with parameters -t sample1.bam -g hs -f BAMPE --broad -n sample1 --keep-dup=all --ourdir.
Data quality	We confirmed that quality control results were good following the ENCODE guideline and reads were converted to bigwig files. We observed clear peaks with inspection on Integrated Genomics Viewer (Broad Institute) and there was high consistency.
Software	Cutadapt (v1.12), Bowtie2 (v2.2.9), SAMtools (v1.3), DeepTools (v3.5.1), R package ChIPseeker (v1.38.0), R package ClusterProfiler (v3.18.0) , MACS2 (v2.2.6) , picard-MarkDuplicates (v1.119).

Flow Cytometry

Plots

Confirm that:

☒ The axis labels state the marker and fluorochrome used (e.g. CD4-FITC).

☒ The axis scales are clearly visible. Include numbers along axes only for bottom left plot of group (a 'group' is an analysis of identical markers).

☒ All plots are contour plots with outliers or pseudocolor plots.

☒ A numerical value for number of cells or percentage (with statistics) is provided.

Methodology

Sample preparation	The hES cells were digested as single cells by accutase and collected for the further procedures. Fixation buffer (BD Biosciences) was used to fix these cells at room temperature for 30 minutes. After washed once with PBS, these cells were permeated in perm/wash buffer (BD Biosciences) at 4 °C for 15 minutes. After that, these cells were incubated with corresponding primary antibodies at 37 °C for 30 minutes. After washed once with PBS, these cells were incubated with corresponding secondary antibodies at 37 °C for 30 minutes. These cells were washed once with PBS and re-suspended with PBS. Then, these samples were detected by Cytoflex (Beckman).<br>The detail information of FACS analysis for EdU assay, EU assay cell cycle and apoptosis assay were described in methods, Please see methods.
Instrument	These samples were analyzed with Cytoflex (Beckman).

Software	These data were analyzed with FlowJo.
Cell population abundance	50,000 cells.
Gating strategy	Cell debris were excluded by FSC-A/SSC-A plot and singlets were gated by SSC-A/SSC-W plot. The gates for positive cells were determined by non staining cells.

☒ Tick this box to confirm that a figure exemplifying the gating strategy is provided in the Supplementary Information.
