# Supplementary figures and images for: METTL3/METTL14 maintain human nucleoli integrity by mediating SUV39H1/H2 degradation
Source: Nat Commun. 2024 Aug 21;15:7186. doi: 10.1038/s41467-024-51742-7 (PMC11339338; doi:10.1038/s41467-024-51742-7)

Fig. 1a

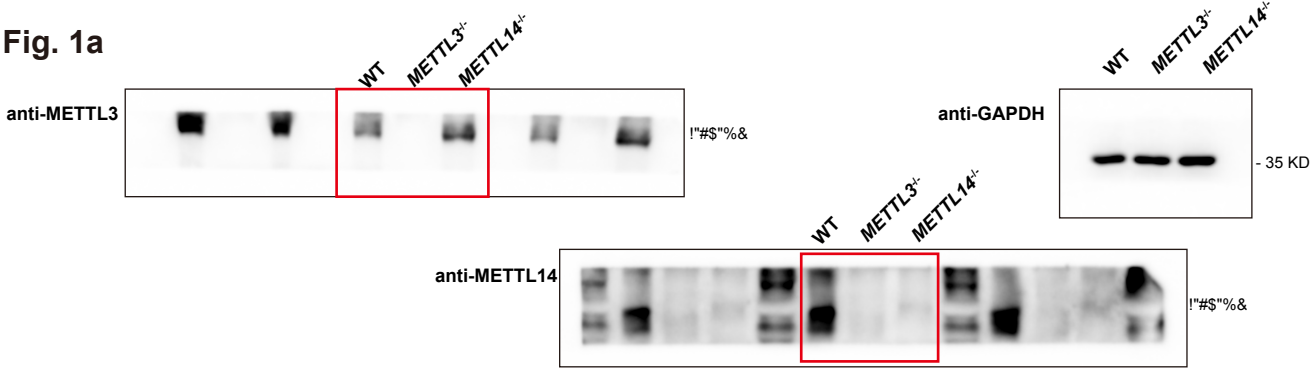

Fig. 1i

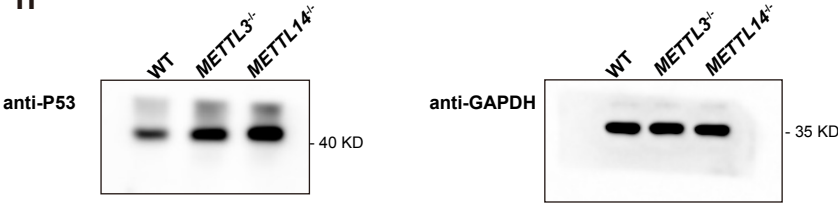

Fig. 3a

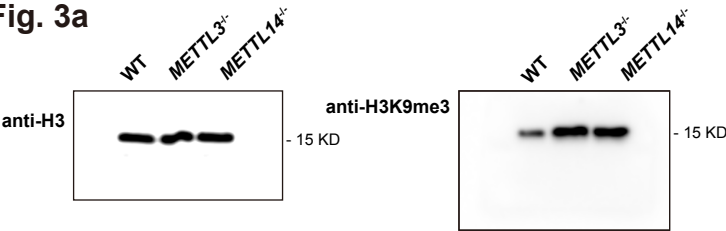

Fig. 3c

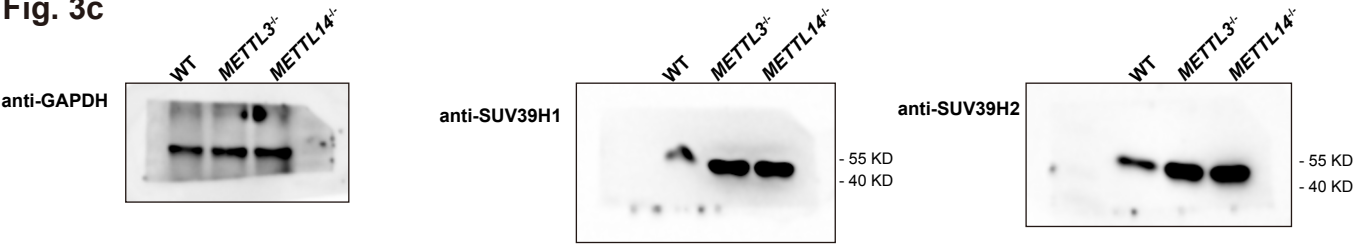

Fig. 5f

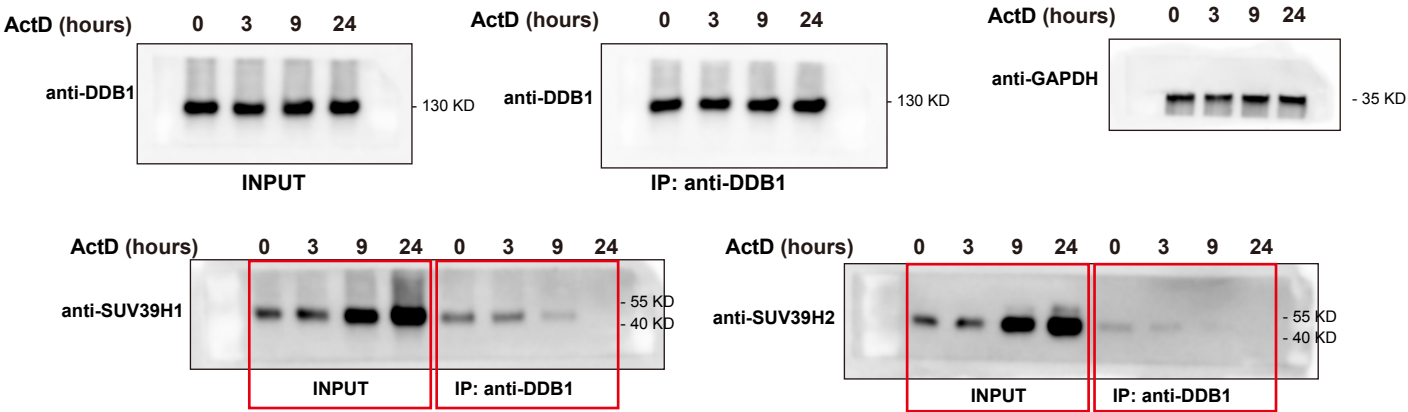

**Fig. 5b**

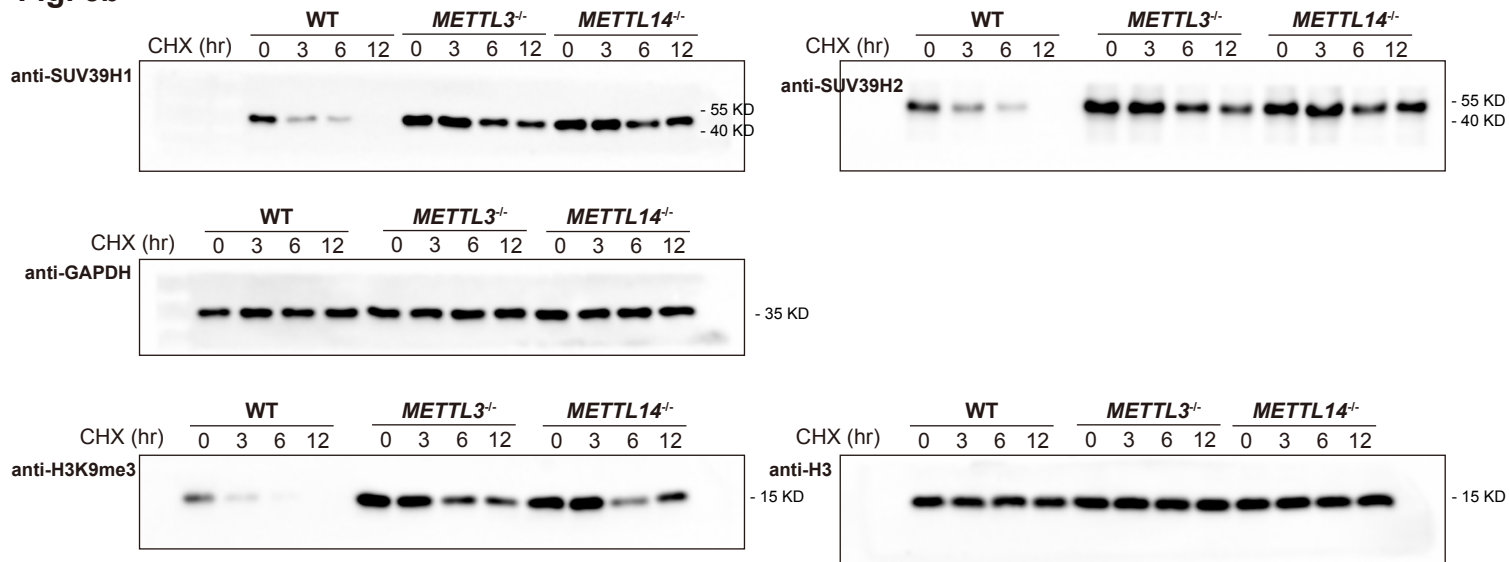

**Fig. 5c**

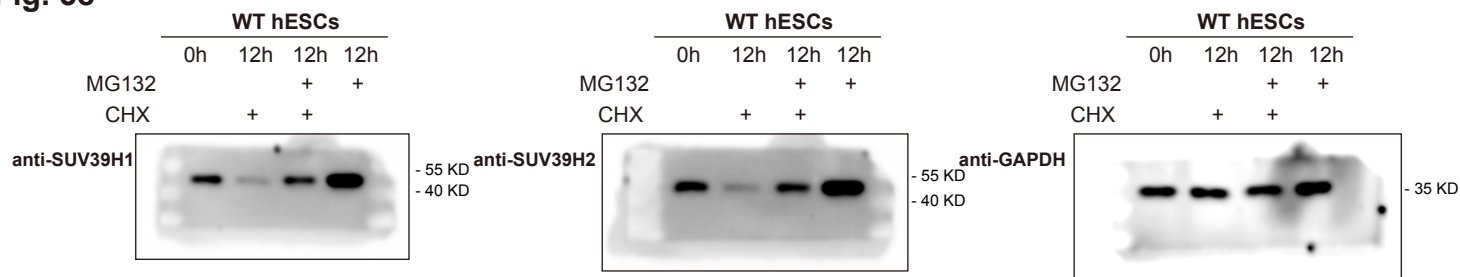

**Fig. 5d**

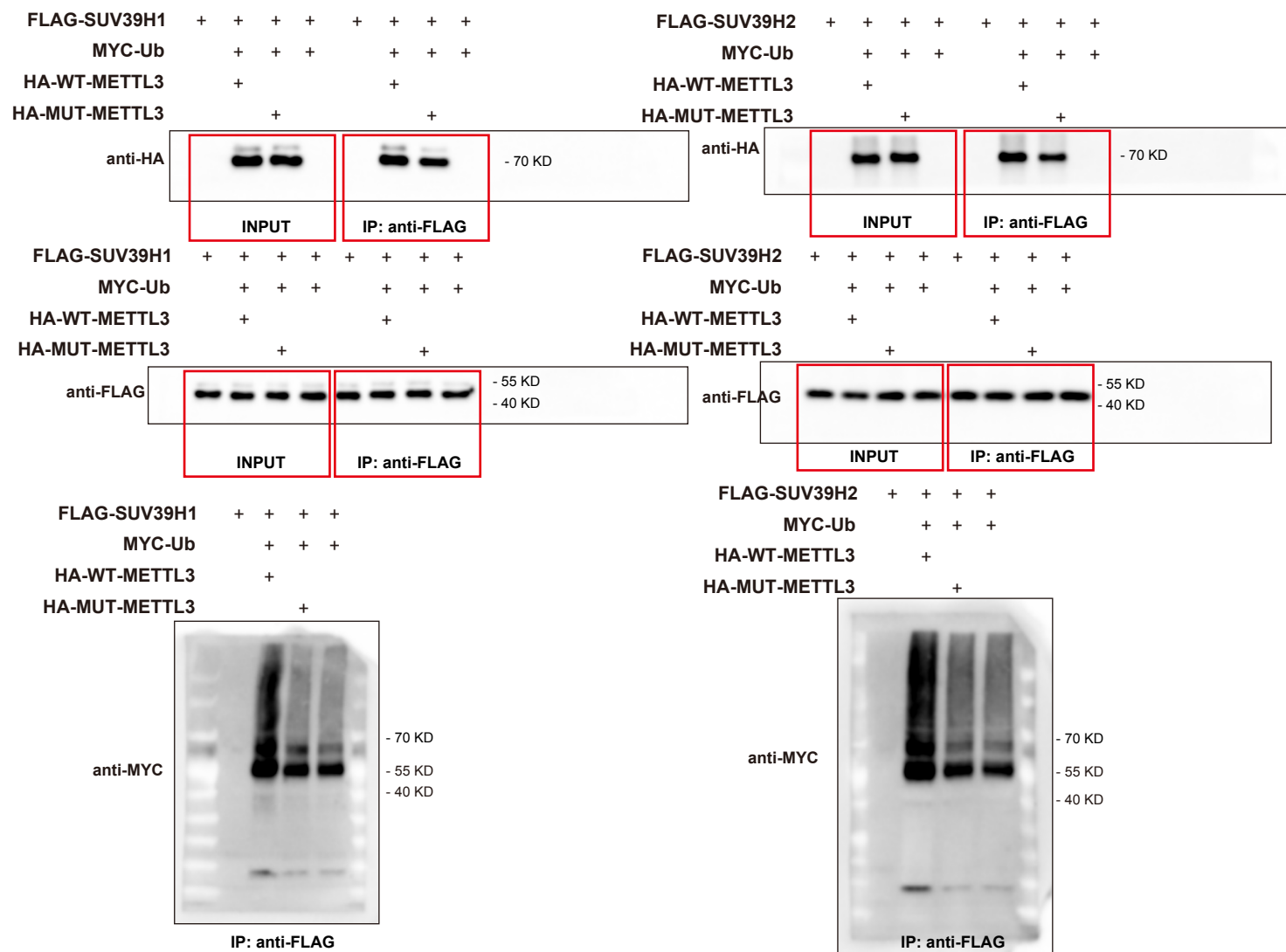

Fig. 5e

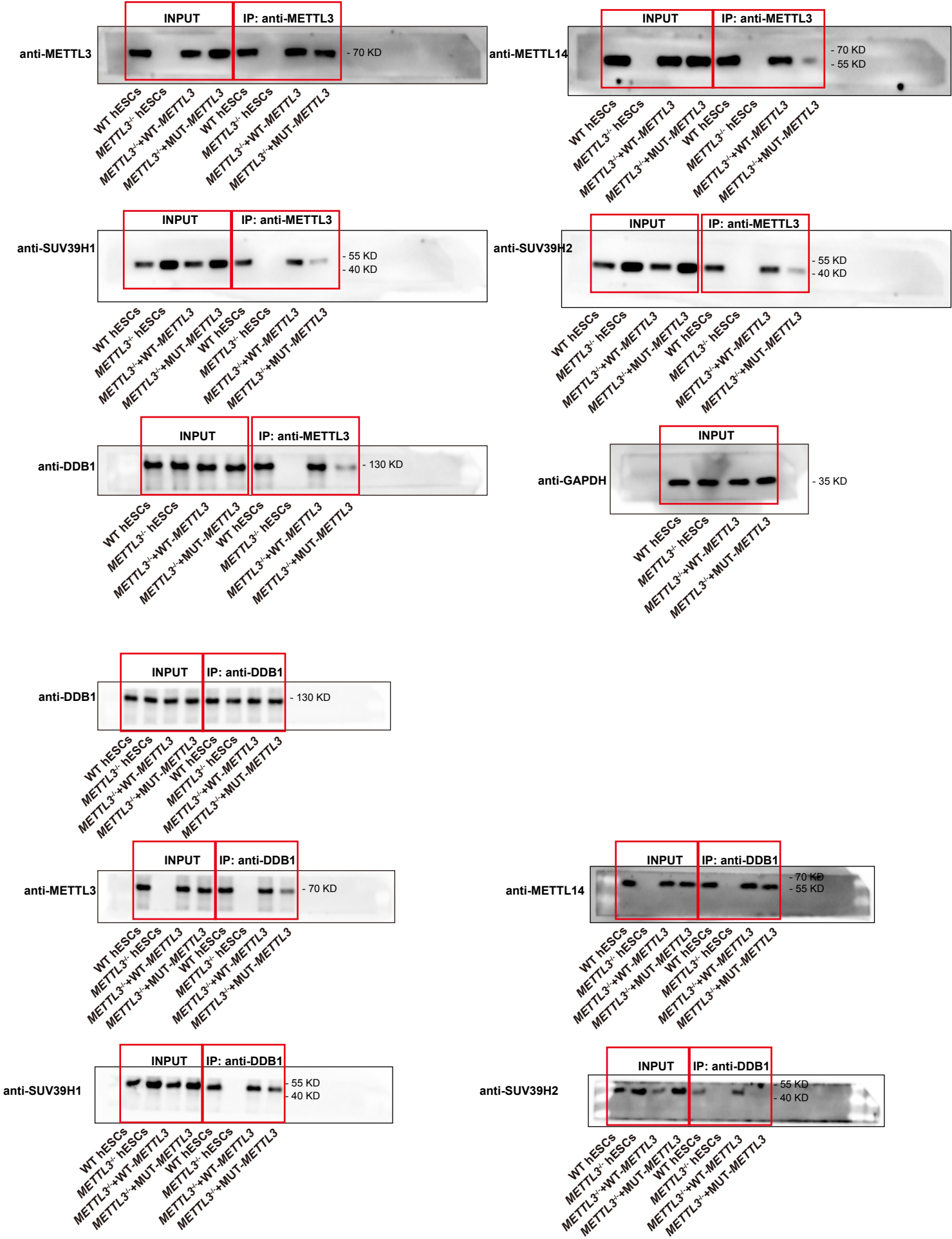

Supplement: Supplementary file 4 — Source Data [file 41467_2024_51742_MOESM4_ESM.zip › Source Data/Original Blots for Figures.pdf]
